# Identification of renal stem cells in zebrafish

**DOI:** 10.1126/sciadv.adx5296

**Published:** 2025-08-22

**Authors:** Ting Yu, Xiaoliang Liu, Xiaoqin Tan, Yunfeng Zhang, Zhongwei He, Wenmin Yang, Tingting Tian, Yan Li, Jinghong Zhao, Chi Liu

**Affiliations:** ^1^ Department of Nephrology, Chongqing Key Laboratory of Prevention and Treatment of Kidney Disease, Chongqing Clinical Research Center of Kidney and Urology Diseases, Xinqiao Hospital, Army Medical University (Third Military Medical University), Chongqing 400037, China.; ^2^ Department of Respiratory and Critical Care Medicine, Xinqiao Hospital, Army Medical University (Third Military Medical University), Chongqing 400037, China.

## Abstract

Renal stem cells (RSC) hold great promise as kidney disease regenerative therapies. However, RSCs capable of regenerating de novo nephrons remain unidentified in vertebrates. Therefore, this study aimed to identify RSCs in zebrafish. Single-cell RNA sequencing revealed *eya2*, *pax2a*, and *six2a* as primary markers of zebrafish RSCs. Real-time imaging demonstrated that RSCs originated from *eya2*-positive mesenchymal cells. Notably, photoconversion-based lineage tracing and serial transplantation assays revealed a unique RSC renewal process, characterized by a differentiation-proliferation-dedifferentiation mode. This process generates nephrons and nascent RSCs concurrently. In addition, precise Wnt signaling is key for RSC renewal and differentiation balance and directly activates *eya2* expression to initiate renewal. This discovery establishes a foundation for the advancement of stem cell therapies for kidney diseases.

## Teaser

Zebrafish renal stem cells can continuously renew and regenerate nephrons via transplantation.

## INTRODUCTION

Numerous human kidney diseases are caused by a loss of nephrons (*1*, *2*), the functional units of the kidney responsible for filtering blood and producing urine. However, mammalian renal progenitor cells (RPCs) that are responsible for nephrogenesis can typically undergo only a limited number of self-renewal cycles (*3*, *4*). These RPCs vanish around birth, limiting nephron repair to a partial extent in humans and impeding the regeneration of new nephrons (*5*–*7*). Therefore, identifying renal stem cells (RSCs) capable of continuous renewal and regeneration of nephrons in adult kidneys holds great promise for the development of new treatments. However, the search for such RSCs has been a longstanding and critical challenge in the field.

Vertebrate kidney development progresses through three stages: pronephros, mesonephros, and metanephros (*8*, *9*). The pronephros, the most primitive, comprises two nephrons. The mesonephros, formed by adding nephrons to pronephric tubules, functions during embryogenesis, persisting in adult zebrafish while regressing in mammals. The metanephros, arising from ureteric bud and metanephric mesenchyme interactions, forms the definitive mammalian kidney (*9*). Despite these variations, the nephron structure remains evolutionarily conserved between zebrafish and mammals (*8*). Zebrafish exhibit a robust capacity to regenerate nephrons throughout their entire life span (*10*). The kidneys of adult zebrafish are composed of approximately 600 nephrons (*11*). In the event of acute kidney injury (AKI), which results in the loss of most nephrons, zebrafish can regenerate a substantial number of nephrons within 15 days, effectively restoring renal function (*11*). RSCs with continuous renewal capacity have been hypothesized to exist in zebrafish kidneys. A previous study (*11*), along with our research (*12*, *13*), revealed that *lhx1a*-positive (*lhx1a*^+^) cell aggregates generate nephrons during kidney development and regeneration, suggesting a close association between RSCs and these cell aggregates. However, it remains unclear whether RSCs reside within the *lhx1a*^+^ cell aggregates or represent precursor cells that give rise to these aggregates. The molecular markers of RSCs have also not yet been defined. In addition, the mechanisms underlying RSC renewal have not yet been explored. Addressing these questions is essential for understanding the fundamental properties of RSCs.

Here, we performed single-cell RNA sequencing (scRNA-seq) on adult zebrafish kidneys postinjury and identified an RSC population marked by *eya2*, *pax2a*, and *six2a* expression. Real-time tracking revealed that these RSCs originated from *eya2*-positive mesenchymal cells at 46 hours post-fertilization (hpf). Photoconversion-based lineage tracing revealed a unique renewal mode: multiple RSCs form a cell aggregate through the mesenchymal-epithelial transition (MET) and begin rapid proliferation. The cells at the bases of these aggregates form nephrons, whereas the cells at the apices, which highly express *eya2* and *six2a*, detach through epithelial-mesenchymal transition (EMT) and generate new RSCs. Serial transplantation assays in adult kidneys further confirmed the continuous renewal and differentiation abilities of RSCs. Mechanistic studies revealed that Eya2, which interacts with Six1b and is regulated by Wnt signaling, is critical for RSC renewal. Our research delineates the key characteristics of RSC generation, differentiation, and renewal.

## RESULTS

### scRNA-seq revealed RSCs

**Fig. 1.**
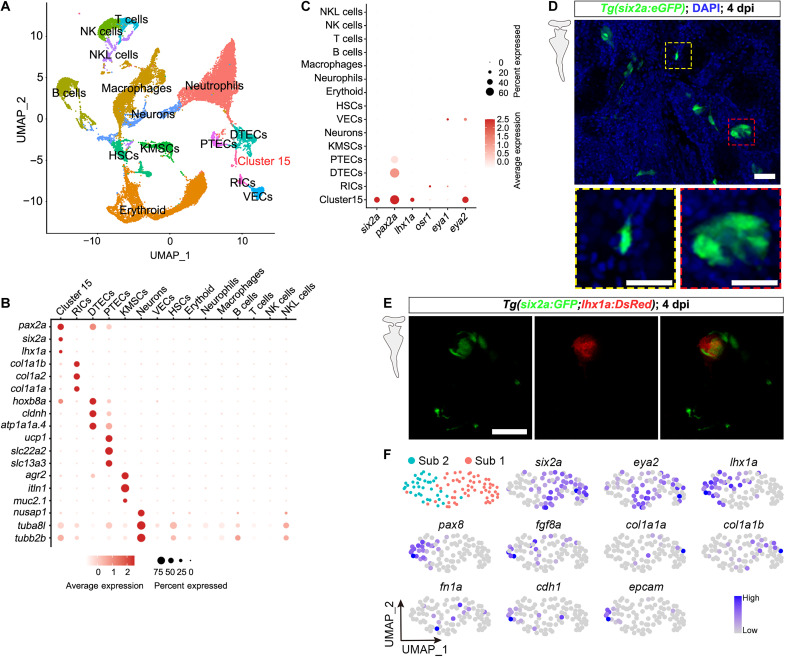
Identification of potential zebrafish RSC clusters via scRNA-seq analysis of injured kidneys. (**A**) Uniform Manifold Approximation and Projection (UMAP) plot analysis identified a previously undefined cell cluster (cluster 15) in the zebrafish kidney. (**B**) Dot plot displaying the relative expression of markers for various kidney cell clusters. Cluster 15 expresses markers associated with mammalian RPCs. (**C**) Dot plot showing the relative expression of *six2a*, *pax2a*, *lhx1a*, *osr1*, *eya1*, and *eya2* across kidney cell clusters. All these genes, except *eya1* and *osr1*, are highly expressed in cluster 15. (**D**) Confocal image of adult *Tg(six2a:eGFP)* kidneys at 4 dpi. The red dashed box outlines a cell aggregate, while the yellow dashed box highlights a dendritically shaped mesenchymal cell. The grayscale image on the left indicates that the image was captured in the adult kidney. (**E**) Confocal images of adult *Tg(six2a:eGFP;lhx1a:DsRed)* kidneys at 4 dpi, showing numerous *six2a*^+^ cells that are not labeled by *lhx1a*. (**F**) UMAP plot showing the identification of RSC subtypes following subclustering of cluster 15 from (A). UMAP feature plots indicate that subpopulation 1 exhibits high expression of *six2a*, *eya2*, *col1a1a*, *col1a1b*, and *fn1a*, whereas subpopulation 2 is characterized by high expression of *lhx1a*, *pax8*, *fgf8a*, *cdh1*, and *epcam*. NK, natural killer; VECs, vascular endothelial cells; DTECs, distal tubular epithelial cells; HSCs, hematopoietic stem cells; KMSCs, kidney mucin–secreting cells; RICs, renal interstitial cells. Scale bars, 50 μm [(D) and (E)]. PTECs, proximal tubular epithelial cells; NKL, natural killer T-like cells.

In this study, we used scRNA-seq to identify potential RSC clusters during kidney regeneration in zebrafish. To induce AKI, we injected gentamicin (Gent; 2.7 μg/μl, 20 μl per fish) intraperitoneally into 12 randomly selected adult zebrafish (*11*, *14*) and collected their kidneys at 4 days postinjury (4 dpi). Through scRNA-seq of these kidneys, we obtained 31,008 cells that passed quality control and were included in subsequent analysis. Initial analysis of these cells revealed 15 distinct cell clusters (Fig. 1A), one of which (cluster 15) showed high expression of mammalian RPC marker gene homologs (*9*, *15*–*18*), including *six2a*, *pax2a*, and *lhx1a* (Fig. 1B and table S1). Consequently, we hypothesized that cluster 15 represents zebrafish RSCs. However, the expression of the key mammalian RPC marker homologs *osr1* and *eya1* (*9*, *15*, *19*, *20*) was barely detectable (Fig. 1C). This suggests differences between zebrafish RSCs and mammalian RPCs.

Six2, the primary marker gene in mammalian RPCs, functions autonomously within cells to sustain progenitor cell status (*17*). To confirm the identity of cluster 15, we generated a reporter line to track *six2a*-expressing cells in zebrafish (fig. S1). Using clustered regularly interspaced short palindromic repeats/CRISPR-associated protein 9 (CRISPR-Cas9), we engineered a *TgKI(six2a:p2a-Gal4-VP16)* reporter line by incorporating a *p2a-Gal4-VP16* cassette into the 3′ end of the *six2a* coding sequence (fig. S1A) (*21*). The Gal4-UAS system, which controls gene expression, allows Gal4 to activate genes downstream of the UAS (upstream activation sequence) (*22*). By crossing this line with *Tg(UAS:eGFP)*, we generated *Tg(six2a:p2a-Gal4-VP16;UAS:eGFP)*, abbreviated as *Tg(six2a:eGFP)* (fig. S1C). Morphological analysis revealed a substantial presence of dendritically shaped mesenchymal cells and spherical cell aggregates expressing *six2a* in the injured kidneys of *Tg(six2a:eGFP)* adult fish at 4 dpi (Fig. 1D). Furthermore, analysis of the hybrid transgenic line *Tg(lhx1a:DsRed;six2a:eGFP)* revealed the presence of *lhx1a*^+^ cell aggregates (Fig. 1E), as previously reported (*11*), along with surrounding individual mesenchymal cells in the kidney. Quantitative analysis of fluorescence signals indicated that most *lhx1a*^+^ cells were also *six2a*^+^ at 4 dpi in adult kidneys. However, more than 90% of *six2a*^+^ mesenchymal cells lacked *lhx1a* expression (*n* = 120 cells from six fish) (Fig. 1E), highlighting the heterogeneity among *six2a*^+^ kidney mesenchymal cells.

Therefore, we further analyzed cluster 15 and found that it could be subdivided into two distinct cell subpopulations (Fig. 1F and table S2). Subpopulation 1, marked by high levels of *six2a* and *eya2*, may represent zebrafish RSCs, given the expression of key mammalian RPC markers (Fig. 1F). In contrast, subpopulation 2 expressed the genes *lhx1a*, *pax8*, and *fgf8a* (Fig. 1F), which are involved in RPC aggregation and differentiation (*9*, *11*, *15*, *23*, *24*), suggesting that these cells may represent components of aggregates derived from RSCs. Furthermore, an analysis of markers for mesenchymal and epithelial cells revealed that subpopulation 1 exhibited higher expression of mesenchymal markers such as *col1a1a*, *col1a1b*, and *fn1a* (*25*, *26*), whereas subpopulation 2 showed elevated expression of epithelial markers, including *cdh1* and *epcam* (Fig. 1F) (*27*). These findings indicate that subpopulation 1 likely has mesenchymal characteristics, while subpopulation 2 exhibits epithelial traits. This is consistent with the observation of *six2a*-labeled mesenchymal cells and cell aggregates in the kidney (Fig. 1, D and E). However, these conclusions require further validation during kidney development in zebrafish.

### Real-time observation of RSC generation

**Fig. 2.**
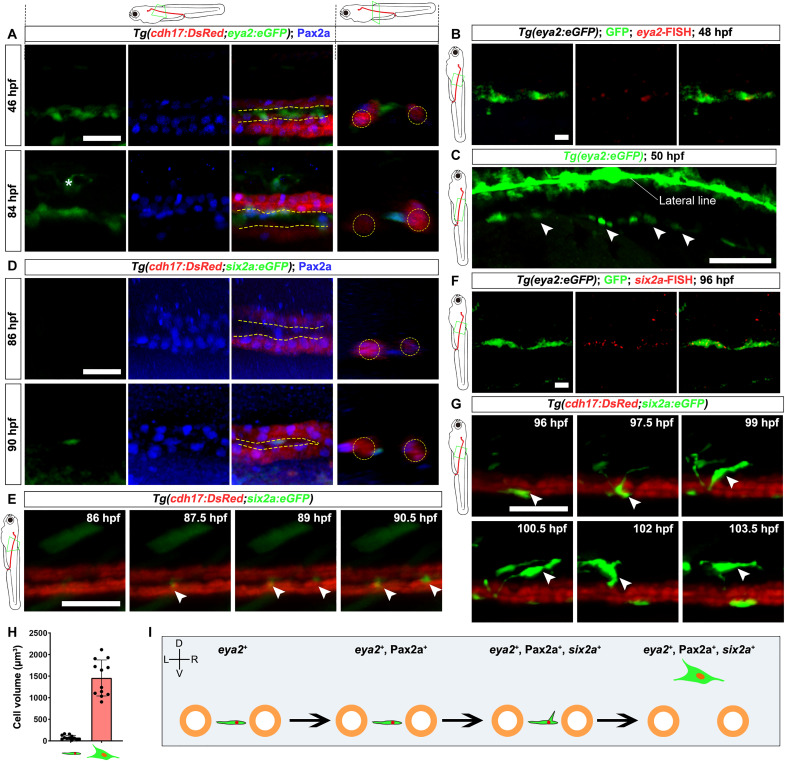
RSC generation process. (**A**) Confocal images of Pax2a immunofluorescence in *Tg(cdh17:DsRed;eya2:eGFP)* zebrafish show that *eya2*^+^ cells emerge at 46 hpf, and these cells start expressing Pax2a by 84 hpf. *cdh17:DsRed* labels renal tubular epithelial cells. The asterisk indicates other *eya2:eGFP*^+^ cell types that have yet to be identified. The yellow dashed lines indicate the intermediate region between the two renal tubules. The line diagram at the top indicates that the images were captured from zebrafish embryos. The red lines represent the renal tubules, while the green box indicates the region and orientation of image acquisition. (**B**) Combination of *eya2*-FISH and GFP immunofluorescence in *Tg(eya2:eGFP)* zebrafish at 48 hpf. (**C**) Confocal image showing *Tg(eya2:eGFP)* zebrafish at 50 hpf. Arrowheads indicate RSCs arranged in a cord-like structure. The structure above it is the lateral line. (**D**) Confocal images of Pax2a immunofluorescence in *Tg(cdh17:DsRed;six2a:eGFP)* zebrafish at 86 and 90 hpf, demonstrating that *six2a* is first expressed at 90 hpf. (**E**) Time-lapse observation of *Tg(six2a:eGFP;cdh17:DsRed)* zebrafish from 86 to 90.5 hpf. Arrowheads indicate *six2a:eGFP*^+^ RSCs. (**F**) Combination of *six2a*-FISH and GFP immunofluorescence in *Tg(eya2:eGFP)* zebrafish at 96 hpf. (**G**) Time-lapse observation of *Tg(cdh17:DsRed;six2a:eGFP)* zebrafish from 96 to 103.5 hpf. Arrowheads indicate *six2a:eGFP*^+^ RSC. (**H**) The cell volume of *six2a:eGFP*^+^ RSCs in (G) (96 and 103.5 hpf) was measured using ImageJ (*n* = 12 cells from three fish). (**I**) Graphical abstract summarizing RSC generation. A subset of *eya2*^+^ mesenchymal cells begins to express Pax2a at approximately 84 hpf, followed by the expression of *six2a*, which facilitates the maturation of RSCs. Yellow circles represent cross sections of the pronephric tubules, and green cells denote RSCs. Axial orientation is shown in the top left corner, indicating the dorsal (D)–ventral (V) and left (L)–right (R) axes. Scale bars, 50 μm [(A) to (G)].

To track the formation process of RSCs, we first needed to identify a marker gene that could effectively label early RSCs. RPCs in mammals originate from mesenchymal cells in the nephrogenic cord marked by *Osr1*, *Lhx1*, and *Eya1* (*9*, *15*, *18*–*20*). Thus, we used the technique of generating the *Tg(six2a:eGFP)* transgenic line and separately developed transgenic lines that accurately represent the expression of these three gene homologs in zebrafish: *osr1*, *lhx1a*, and *eya1* (figs. S2 to S4). Using these lines, we did not observe any enhanced green fluorescent protein (eGFP)^+^ cells between the pronephric ducts before 48 hpf (fig. S5). Eya1 functions at the top of the regulatory network to maintain the mammalian RPC population throughout kidney development (*20*). However, scRNA-seq revealed that *eya1* expression in cluster 15 cells was barely detectable. Instead, we detected a high expression of *eya2* (Fig. 1C), a gene within the *eya* family with a function similar to that of *eya1* (*28*). Using the *Tg(eya2:eGFP)* transgenic line constructed according to the aforementioned methods (fig. S6), we detected *eya2:eGFP*^+^ cells between pronephric ducts at 46 hpf (Fig. 2A). Using fluorescence in situ hybridization (FISH), we confirmed the initiation of *eya2* expression in these cells (Fig. 2B). These *eya2*^+^ cells, which were small and flat, lined up between the pronephric ducts in a cord-like structure (Fig. 2, A and C).

Immunofluorescence with Pax2a, which marks RPCs and renal tubules (*16*, *23*), revealed that a subset of *eya2:eGFP*^+^ cells began expressing Pax2a from 84 hpf onward (Fig. 2A). At 90 hpf, these Pax2a^+^ cells began to express *six2a:eGFP* (Fig. 2D), and this signal gradually intensified over time (Fig. 2E and movies S1 and S2). To further confirm the identity of these cells, we performed *six2a* FISH on 96 hpf *Tg(eya2:eGFP)* embryos. The results showed that *six2a*^+^ cells located between the pronephric tubules were also *eya2*^+^ (Fig. 2F). Starting at 97 hpf, the *six2a:eGFP*^+^ cells began to extend pseudopodia and migrate dorsally, ceasing ~20 μm above the pronephric ducts (Fig. 2G and movie S3). Concurrently, the cell volume expanded ~18.5-fold (Fig. 2, G and H). These data indicate that a subset of *eya2*^+^ mesenchymal cells began to express Pax2a by approximately 84 hpf, followed by the initiation of *six2a* expression and the maturation of RSCs (Fig. 2I).

### Differentiation and renewal of RSCs

**Fig. 3.**
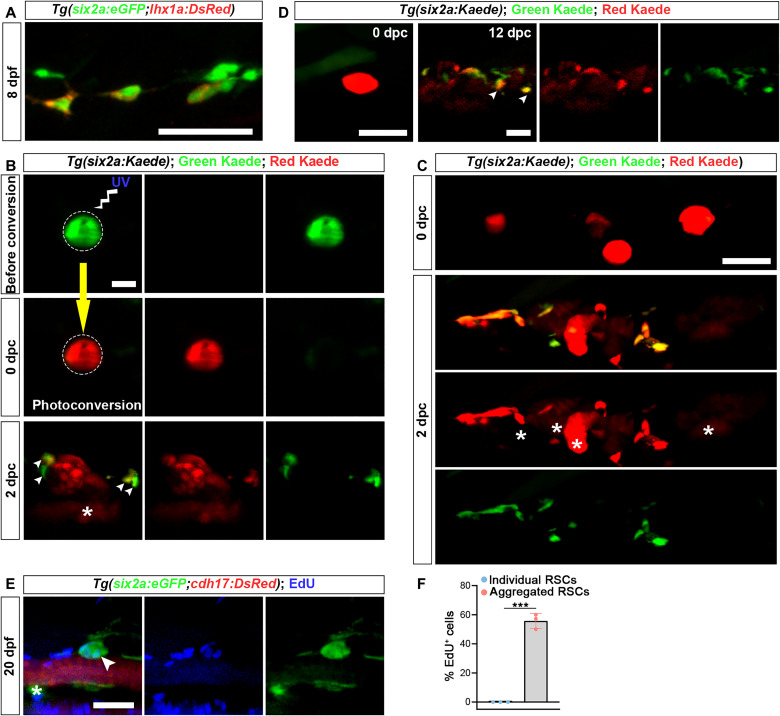
Renewal of RSCs. (**A**) Confocal images of *Tg(six2a:eGFP;lhx1a:DsRed)* zebrafish at 8 dpf show that *six2a:eGFP*^+^ RSCs begin to express *lhx1a* at this stage. (**B**) Confocal images showing *six2a:Kaede*^+^ RSC aggregate before and after photoconversion. Only the circled area was photoconverted. The asterisk indicates red Kaede^+^ nascent nephrons, while arrowheads indicate nascent RSCs. dpc, days postphotoconversion. (**C**) In individuals where all RSCs that had formed aggregates were photoconverted, nascent RSCs exhibited red fluorescence at 2 dpc. Asterisks indicate red Kaede^+^ nascent nephrons. (**D**) Long-term photoconversion-based lineage tracing revealed that nascent RSCs were capable of forming cell aggregates in newly formed nephrons at 12 dpc. Arrowheads indicate newly formed RSC aggregates. (**E**) EdU assay of *Tg(six2a:eGFP;cdh17:DsRed)* zebrafish at 20 dpf. EdU incorporation is primarily observed in RSC aggregate (arrowhead) rather than in individual RSCs (asterisk). (**F**) Quantification of EdU^+^ RSCs from (E), showing the proportion of EdU^+^ cells within aggregates or as individual RSCs. *n* = 3 fish, with 30 cells analyzed per group within each fish. Scale bars, 50 μm [(A) to (E)].

Subsequently, as more *six2a*^+^ cells migrated above and aligned along the pronephric tubules, they began to aggregate (fig. S7A). At approximately 8 dpf, using the *Tg(lhx1a:DsRed;six2a:eGFP)* hybrid line, we observed the onset of DsRed expression in *six2a*^+^ RSCs (Fig. 3A), confirming that the *lhx1a*^+^ cells identified in previous studies (*11*) originate from *six2a*^+^ cells. Consistent with prior findings (*11*), these *lhx1a*^+^ cells begin to form cell aggregates and undergo differentiation and proliferation, ultimately giving rise to renal vesicles (RVs). The RVs then elongate, forming new nephrons (fig. S7, A and B). To further confirm that *six2a*^+^ cells form cell aggregates, we performed photoconversion-based lineage tracing using Kaede, a fluorescent protein that shifts from green to red under ultraviolet light, making it ideal for cell lineage analysis (*29*). By crossing *TgKI(six2a-p2a-Gal4-VP16)* with *Tg(UAS:Kaede)*, we generated the hybrid *Tg(six2a:Kaede)* line, which enabled continuous Kaede expression in RSCs (fig. S7C). At 9 dpf, we photoconverted individual *six2a:Kaede*^+^ cells one at a time and tracked their fate. These cells clustered with other RSCs to form cell aggregates (fig. S7C). Previous studies using real-time tracking analysis and laser ablation have established that *lhx1a*^+^ cell aggregates act as precursors for nephron formation (*11*). In parallel, our research reveals that *six2a*^+^ cells are constituent cells of these aggregates. Thus, *six2a*^+^ cells serve as precursor cells for both aggregate and nephron formation.

To further investigate the differentiation process of cell aggregates, we used the *Tg(six2a:Kaede)* line. By specifically photoconverting one cell aggregate and tracking it, we found that a cell aggregate could give rise to a new nephron and five to seven RSCs (*n* = 6 cell aggregates from six fish) surrounding it (Fig. 3B). Immunofluorescence revealed that red Kaede colocalized with Pax2a and podocin (podocyte marker) (fig. S8A) (*30*), indicating their differentiation into all epithelial cell types of the nephron. Simultaneously, we conducted experiments on individuals in which all the RSCs clustered into cell aggregates and photoconverted all of the aggregates (Fig. 3C). Through tracking, all individual RSCs around the new nephrons displayed red fluorescence (*n* = 4 fish) (Fig. 3C), demonstrating that the newly generated RSCs all originated from cell aggregates. Continuing our tracking, we observed that nascent red fluorescent RSCs could aggregate in the renal tubules of newly formed nephrons, forming RSC aggregates (Fig. 3D). This indicated the capacity of nascent RSCs for ongoing renewal and differentiation.

A hallmark of classical stem cells, including hematopoietic stem cells, is their sustained self-renewal ability (*31*). To investigate this phenomenon in zebrafish RSCs, we used an EdU assay to determine whether RSCs undergo self-renewal or generate subsequent generations through proliferation within cell aggregates. Our analysis revealed no EdU incorporation in individual eGFP^+^ RSCs in juvenile *Tg(six2a:eGFP)* zebrafish, whereas proliferative signals were exclusively detected within RSC aggregates (Fig. 3, E and F). These findings suggest that individual RSCs lack the capacity for self-renewal via proliferation. Given that proliferative activity is restricted to cell aggregates and that these aggregates are capable of generating nascent RSCs, we conclude that RSC renewal occurs within RSC aggregates.

**Fig. 4.**
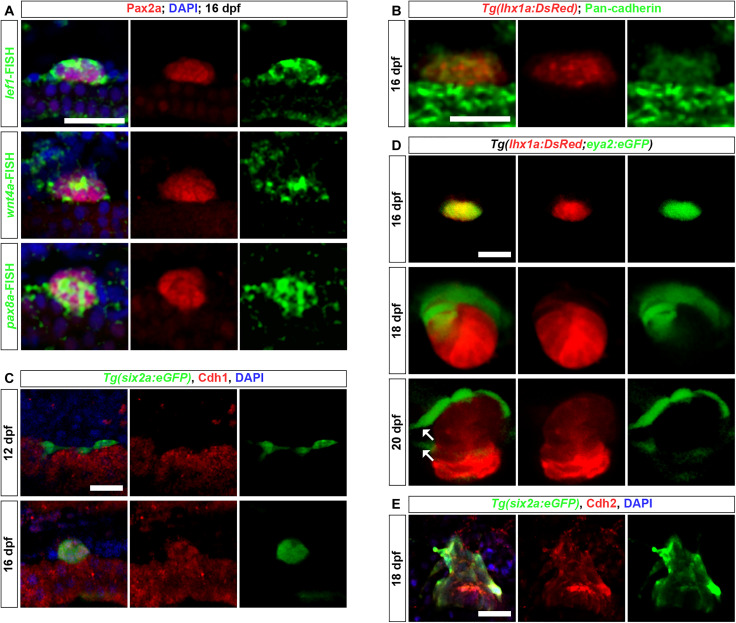
MET and EMT processes during RSC renewal. (**A**) The combination of *lef1*, *wnt4a*, and *pax8* FISH with Pax2a immunofluorescence shows high expression of *lef1*, *wnt4a*, and *pax8* in Pax2a^+^ RSC aggregates at 16 dpf. (**B**) Pan-cadherin immunofluorescence in *Tg(lhx1a:DsRed)* zebrafish at 16 dpf indicates that RSCs begin to establish cell junctions after aggregation. (**C**) Cdh1 immunofluorescence in *Tg(six2a:eGFP)* zebrafish shows that RSC aggregates begin to express the MET marker Cdh1 after aggregation. (**D**) Confocal images of *Tg(lhx1a:DsRed;eya2:eGFP)* zebrafish at 16, 18, and 20 dpf. Arrows indicate newly formed individual RSCs. (**E**) Cdh2 immunofluorescence in *Tg(six2a:eGFP)* zebrafish shows that RSC aggregates highly express the EMT marker Cdh2 during the generation of new RSCs. Scale bars, 50 μm [(A) to (E)]. DAPI, 4′,6-diamidino-2-phenylindole.

To further elucidate the RSC renewal process, we performed FISH on cell aggregates before RV formation to detect the differentiation marker genes *lef1*, *wnt4a*, and *pax8* in RSCs (*11*, *13*, *32*). We confirmed that, following aggregation, all RSCs initiated the expression of these genes (Fig. 4A), indicating the onset of their differentiation. Using immunofluorescence for Pan-cadherin (*13*), we identified cell-cell junctions within the aggregates (Fig. 4B). In addition, immunofluorescence staining revealed a significant increase in the MET marker Cdh1 (*33*) during the formation of cell aggregates by RSCs (Fig. 4C), suggesting that RSCs undergo MET during differentiation. Cells within aggregates can proliferate rapidly (*13*, *32*). These results indicate that RSCs undergo differentiation and proliferation after aggregation.

Subsequently, we crossed *Tg(six2a:eGFP)* or *Tg(eya2:eGFP)* lines with *Tg(lhx1a:DsRed)* to study RSC renewal in these hybrids. During the initial formation of cell aggregates, both *eya2:eGFP* and *six2a:eGFP* were highly expressed throughout the cell aggregate (Fig. 4D and fig. S8B). As development progressed, the cells at the base of the aggregate maintained strong *lhx1a:DsRed* expression and diminished eGFP expression. Conversely, the apex of the aggregate showed strong eGFP and minimal DsRed expression. As the cell aggregates elongated, individual cells positive for *eya2:eGFP* or *six2a:eGFP* detached from the apex of the cell aggregates and forming nascent RSCs (Fig. 4D and fig. S8B). To assess whether nascent RSCs coexpress *eya2* and *six2a*, we crossed *Tg(pax2a:DsRed)* fish with *Tg(six2a:eGFP)* or *Tg(eya2:eGFP)* and examined nascent RSCs. All *six2a:eGFP*^+^ RSCs were *pax2a:DsRed*^+^, and all *pax2a:DsRed*^+^ RSCs were *eya2:eGFP*^+^ (fig. S8, C and D), indicating that nascent RSCs are double-positive for *eya2* and *six2a*.

The transformation of differentiating epithelial cells into mesenchymal stem cells indicates that dedifferentiation and EMT may be occurring. To confirm the presence of EMT, we assessed the expression of Cdh2, a well-known EMT marker (*34*). Immunofluorescence studies revealed high levels of Cdh2 in both mature aggregates and detaching RSCs (Fig. 4E), thereby substantiating the occurrence of dedifferentiation and EMT within the aggregates. To investigate whether all apex cells develop into nascent RSCs, we labeled and tracked entire cell aggregates in the *Tg(six2a:Kaede)* line. In ~16.6% of these aggregates (*n* = 12 aggregates from 12 fish), a small subset of apex cells with strong green fluorescence also differentiated into nephron epithelial cells (fig. S8E). This indicates that not all apex cells generate nascent RSCs, although the proportion of such events is relatively low.

### The renewal of RSCs during kidney regeneration

**Fig. 5.**
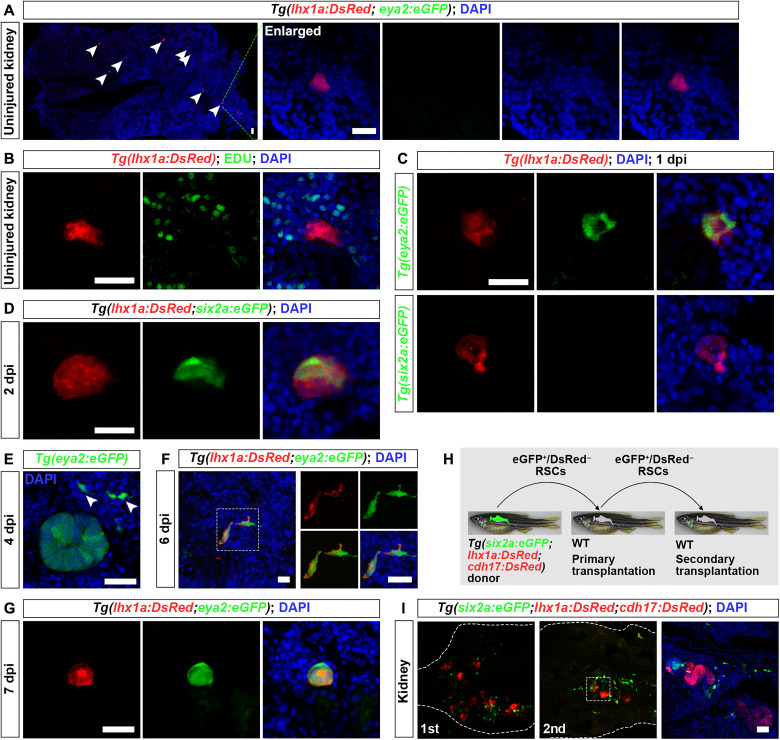
Renewal of RSCs in adult kidneys. (**A**) Confocal images of uninjured adult *Tg(lhx1a:DsRed;eya2:eGFP)* kidney sections showing *lhx1a:DsRed*^+^ RSC aggregates, with the cell aggregate (indicated by arrowheads) magnified for detailed observation. At this stage, RSC aggregates do not express *eya2:eGFP*. (**B**) EdU assay of uninjured *Tg(lhx1a:DsRed)* kidney sections. EdU was administered intraperitoneally every third day until kidney collection for analysis. Results show that most RSC aggregates are not labeled by EdU. (**C**) Confocal images of *Tg(lhx1a:DsRed;eya2:eGFP)* and *Tg(lhx1a:DsRed;six2a:eGFP)* kidney sections at 1 dpi, showing that the aggregates begin to express *eya2:eGFP* but not *six2a:eGFP*. Each RSC aggregate contains approximately 20 cells at this stage. (**D**) Confocal images of *Tg(lhx1a:DsRed;six2a:eGFP)* kidney sections at 2 dpi, showing the appearance of *six2a:eGFP*^+^ cells. By this stage, the RSC aggregates expand to approximately 50 cells. (**E**) Confocal images of *Tg(eya2:eGFP)* kidney sections at 4 dpi, indicating that nascent RSCs emerge. Arrowheads indicate individual RSCs. (**F** and **G**) Confocal images of *Tg(lhx1a:DsRed;eya2:eGFP)* kidney sections at 6 dpi (F) and 7 dpi (G), showing that nascent RSCs begin to express *lhx1a* and aggregate to form new cell aggregates. (**H**) Schematic of the serial transplantation assay. (**I**) Confocal images showing donor-derived nephrons (*cdh17:DsRed*^+^) and RSCs (*six2a:eGFP*^+^) in primary and secondary engrafted recipients. White dashed lines indicate the boundary of the kidney. Scale bars, 50 μm [(A) to (E) and (G) to (I)] and 10 μm (F).

We first monitored RSC renewal using the *Tg(eya2:eGFP;lhx1a:DsRed)* line during adult kidney regeneration (Fig. 5A). In each uninjured kidney, we identified approximately 70 *lhx1a:DsRed^+^* RSC aggregates without *eya2* expression (*n* = 10 fish). By counting the number of 4′,6-diamidino-2-phenylindole–positive nuclei within these aggregates, we found that each consisted of approximately 12 cells [12 ± 2.13 (mean ± SD); *n* = 20 aggregates from five fish] (Fig. 5A). The quiescent state is one of the basic states of adult stem cells, which preserves stem cell renewal and genomic integrity (*35*). To determine whether these aggregates were quiescent, we administered EdU intraperitoneally to uninjured adult fish at 3-day intervals for a total of seven injections. Analysis of the kidneys postinjection revealed that more than 99% of the cells within the aggregates were EdU negative (*n* = 120 cells from 10 aggregates) (Fig. 5B), indicating that they were predominantly in a nonproliferative, quiescent state.

However, upon injury, quiescent cell aggregates began to proliferate and initiated *eya2* expression (Fig. 5C). In injured adult kidneys of *Tg(eya2:eGFP;lhx1a:DsRed)* and *Tg(six2a:eGFP;lhx1a:DsRed)* lines, we observed that most cells initiated *eya2:eGFP* expression within the aggregates at 1 dpi. At this stage, the cell count of each aggregate had increased to approximately 20 [20.4 ± 3.0 (mean ± SD); *n* = 20 aggregates from five fish] (Fig. 5C). Later, as the aggregates expanded, the apex cells within these aggregates began expressing *six2a:eGFP*, and the cell count of the aggregates was approximately 51 [51.2 ± 4.4 (mean ± SD); *n* = 20 aggregates from five fish] (Fig. 5D). These findings indicated that *eya2* reactivation precedes that of *six2a*, suggesting a primary role in the renewal of RSCs. By 4 dpi, a substantial population of *eya2:eGFP*^+^ and *six2a:eGFP*^+^ RSCs had detached from the cell aggregates, giving rise to a new generation of RSCs (Fig. 5E and fig. S9A). Pax2a immunofluorescence revealed that all individual nascent RSCs were *eya2*^+^, *six2a*^+^, and Pax2a^+^ (fig. S9, A and B). This mode of RSC renewal mirrors the mechanism underlying RSC renewal during kidney development. By 6 dpi, nascent RSCs began expressing *lhx1a:DsRed* (Fig. 5F and fig. S9C). By 7 dpi, these nascent RSCs reaggregated to form a new batch of cell aggregates (Fig. 5G and fig. S9D). All cells within these nascent aggregates were *eya2:eGFP*^+^, which differed from the cell aggregates activated from a quiescent state, where only a subset expressed *eya2* (Fig. 5C). This demonstrates that these cell aggregates are formed by nascent RSCs and further supports the sustained renewal and differentiation ability of RSCs.

To further demonstrate the sustained renewal and differentiation capacity of adult RSCs, we conducted serial transplantation experiments (Fig. 5H). RSCs activated at 4 dpi were *six2a^+^*, *eya2^+^*, and *lhx1a*^−^. Therefore, we used a strain obtained by crossing the *Tg(six2a:eGFP)*, *Tg(cdh17:DsRed)*, and *Tg(lhx1a:DsRed)* lines, named *Tg(six2a:eGFP;cdh17:DsRed;lhx1a:DsRed)*, as the donor. In the adult kidneys of this strain, we distinctly identified eGFP^+^ DsRed^−^ RSCs, eGFP^+^ DsRed^+^ RSC aggregates and eGFP^−^ DsRed^+^ nephrons at 4 dpi. After transplanting approximately 50 eGFP^+^ DsRed^−^ RSCs, by day 21 posttransplantation, we detected new nephrons, RSC aggregates, and RSCs in 38% of the irradiated AB-recipient fish kidneys (*n* = 50 fish). In one case, approximately 8 nephrons, 10 RSC aggregates, and 78 RSCs were produced (Fig. 5I). We then performed a second transplantation using RSCs from the transplanted kidneys. This subsequent round also led to new nephrons, RSC aggregates, and RSCs in 25% of kidneys (*n* = 20 fish) (Fig. 5I). These serial transplantation experiments, combined with the lineage tracing experiments mentioned above, demonstrated the ability of RSCs to sustain renewal and differentiation.

### Eya2 regulated RSC renewal through interaction with Six1b

**Fig. 6.**
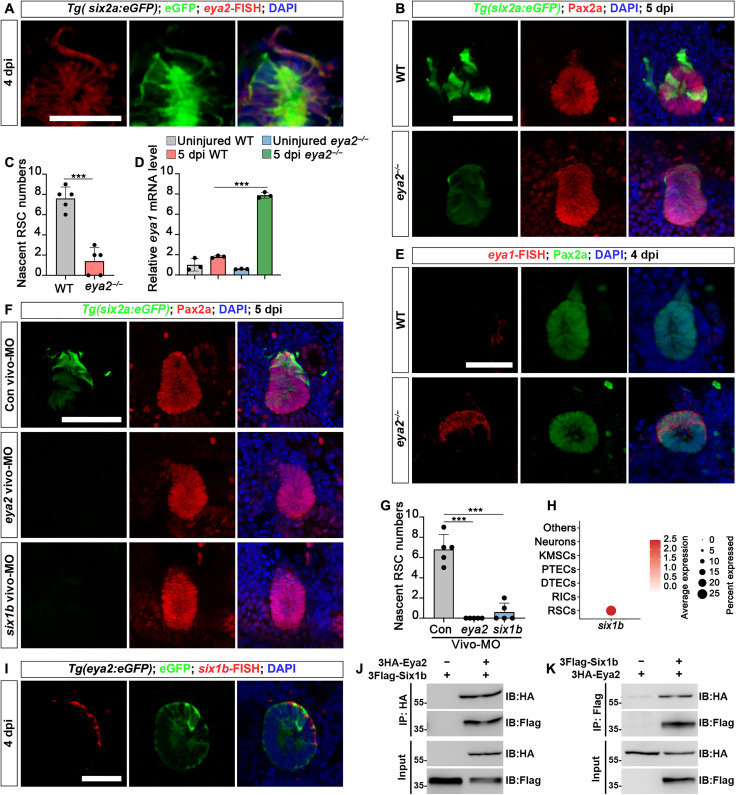
Eya2-Six1b complex regulates RSC renewal. (**A**) Combination of *eya2*-FISH and GFP immunofluorescence in *Tg(six2a:eGFP)* zebrafish at 4 dpi. (**B**) Confocal images of Pax2a immunofluorescence in *Tg(eya2:eGFP)* kidney sections show a significant reduction of nascent RSCs in *eya2*^−/−^ kidneys compared to WT at 5 dpi. (**C**) Quantification of nascent *six2a:eGFP*^+^ RSCs in WT and *eya2*^−/−^ zebrafish kidney sections from (A). *n* = 5 cell aggregates for both groups. (**D**) qRT-PCR analysis of *eya1* expression in WT and *eya2*^−/−^ zebrafish kidneys, both uninjured and at 5 dpi. The data are presented as fold changes relative to *eya1* levels in the uninjured WT. (**E**) Combination of *eya1* FISH and Pax2a immunofluorescence in *eya2*^−/−^ zebrafish at 4 dpi. (**F**) Confocal images of adult *Tg(six2a:eGFP)* kidneys at 5 dpi following administration of control vivo-MO, *eya2* vivo-MO, or *six1b* vivo-MO at −1, 2, and 4 dpi. (**G**) Quantification of the number of *six2a:eGFP*^+^ RSCs around cell aggregates in (F). *n* = 5 cell aggregates for all groups. (**H**) Dot plot showing the relative expression of *six1b* in different kidney cell types. (**I**) At 4 dpi, *six1b* FISH in *Tg(eya2:eGFP)* kidneys demonstrated the coexpression of *eya2* and *six1b* in RSCs. (**J** and **K**) Coimmunoprecipitation assay demonstrating the interaction between Eya2 and Six1b. 3HA-Eya2 and 3Flag-Six1b were coexpressed in HEK293T cells. The data in [(C), (D), and (G)] were analyzed using a two-sided *t* test and are presented as mean values ± SD. ***P < 0.001. Scale bars, 100 μm [(A) to (I)]. IB, immunoblotting.

To validate the contribution of *eya2* to RSC renewal, we first performed *eya2*-FISH in the kidneys of *Tg(six2a:eGFP)* fish at 4 dpi. The results revealed that *eya2* was highly expressed in RSCs (Fig. 6A). Subsequently, we used CRISPR-Cas9 to generate an *eya2* mutant strain. In *eya2*^−/−^ adult kidneys, we observed a marked reduction in RSC renewal at 5 dpi, although renewal was not entirely halted (Fig. 6, B and C). Considering prior findings on genetic compensation by analogous genes in mutants (*36*), we assessed whether *eya1* compensates for *eya2* in *eya2^−/−^* kidneys. Quantitative reverse transcription polymerase chain reaction (qRT-PCR) indicated a substantial increase in *eya1* expression postinjury in *eya2*^−/−^ kidneys compared to its low expression in wild-type (WT) kidneys (Fig. 6D). Similarly, FISH also detected an up-regulation of *eya1* in RSCs within *eya2*^−/−^ kidneys at 4 dpi (Fig. 6E). This suggests genetic compensation by *eya1* in *eya2^−/−^*, although *eya1* did not fully restore RSC renewal efficiency. Because of the absence of genetic compensation in gene knockdown experiments (*36*), we used *eya2* vivo-morpholino (vivo-MO) to knock down the expression of *eya2* in adult *Tg(six2a:eGFP)* kidneys (fig. S10A). This resulted in the inhibition of *six2a:eGFP* reexpression in cell aggregates and renewal of RSCs (Fig. 6, F and G). These findings suggest that *eya2* is essential for RSC renewal.

Eya2 predominantly interacts with Six1 and playing a pivotal role in the development of organs, including the eyes and inner ear (*37*). Nonetheless, *Eya2* expression has not been detected in mammalian RPCs during kidney development (*9*, *15*). This raised the question of whether Eya2 operates via Six1b in zebrafish RSCs. By analyzing scRNA-seq data from adult RSCs, we found high *six1b* expression (Fig. 6H). This expression pattern was further verified using FISH (Fig. 6I). To investigate whether zebrafish Eya2 interacts physically with Six1b, we observed that in human embryonic kidney (HEK) 293T cells, hemagglutinin (HA)–tagged Eya2 coimmunoprecipitated with Flag-tagged Six1b (Fig. 6, J and K), confirming their interaction. In addition, we investigated the involvement of *six1b* in RSC renewal. Knockdown of *six1b* with vivo-MO during kidney regeneration inhibited RSC renewal and *six2a:eGFP* reexpression, mimicking *eya2* deficiency effects (Fig. 6, F and G, and fig. S10B). Six1 activates key genes involved in RPC development such as *Pax2*, *Sall1*, and *Six2* (*38*). This explains how *eya2* regulated the production of *six2a:eGFP*^+^ cells. These results suggest that the Eya2-Six1b interaction regulates RSC renewal.

### Wnt signaling pathway regulated the renewal of RSCs through *eya2*

**Fig. 7.**
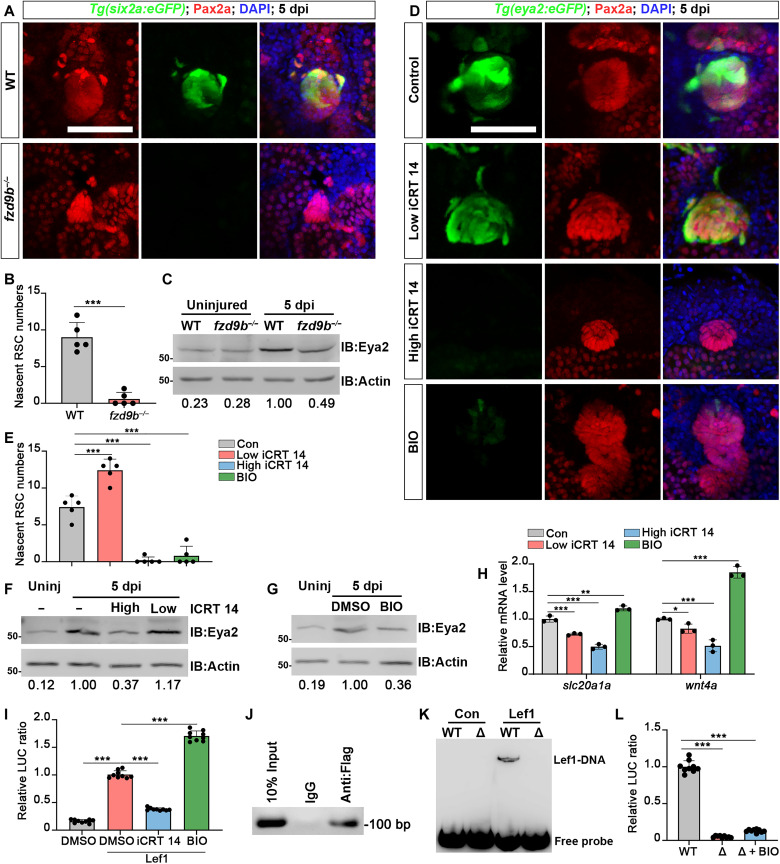
Wnt regulates the renewal of RSCs by directly activating *eya2* expression. (**A**) Pax2a immunofluorescence in *Tg(six2a:eGFP)* kidney sections shows reduced RSC renewal in *fzd9b*^−/−^ zebrafish at 5 dpi. (**B**) Quantification of RSCs generated per aggregate in WT and *fzd9b*^−/−^ kidneys (A, *n* = 5). (**C**) Western blot of Eya2 levels in WT and *fzd9b*^−/−^ kidneys at 5 dpi, normalized to actin and expressed as fold change relative to WT. (**D**) Confocal images of adult *Tg(eya2:eGFP)* kidneys at 5 dpi, treated with Low iCRT 14 (5 μM, 10 μl per fish), high iCRT 14 (80 μM, 10 μl per fish), BIO (10 μM, 10 μl per fish), or dimethyl sulfoxide (DMSO; 0.1%, 10 μl per fish) at 2 and 4 dpi. (**E**) Quantification of the number of RSCs generated per aggregate (D, *n* = 5). (**F** and **G**) Western blot analysis of Eya2 levels in uninjured kidneys and kidneys at 5 dpi after administration (at 2 and 4 dpi) of iCRT 14, BIO, or DMSO. (**H**) qRT-PCR analysis of *slc20a1a* and *wnt4a* expression in kidneys at 5 dpi after administration (at 2 and 4 dpi) of low iCRT 14, high iCRT 14, BIO, or DMSO, expressed as fold change versus WT. (**I**) Relative *eya2* promoter–driven luciferase (LUC) activity measured in HEK293T cells and *lef1*-overexpressed HEK293T cells after administration of iCRT 14 (40 μM), BIO (0.6 μM), or 0.1% DMSO. (**J**) Chromatin immunoprecipitation followed by polymerase chain reaction (ChIP-PCR) confirming Lef1 binding to the predicted genomic site in zebrafish embryos (*n* = 9). (**K**) EMSA shows Lef1 binds *eya2* promoter sequence 5′-CATCAAAG-3′ (Δ: sequence deleted). (**L**) Relative *eya2* promoter–driven LUC activity measured in *lef1*-overexpressed HEK293T cells (*n* = 9). The data in [(B), (E), (H), (I), and (L)] were analyzed by a two-sided *t* test, mean ± SD; *P < 0.05, **P < 0.01, and ***P < 0.001. Scale bars, 100 μm [(A) and (D)].

The Wnt signaling pathway is crucial for nephron development and regeneration, and the differentiation of both zebrafish RSCs and mammalian RPCs is directly regulated by key factors such as *Wnt4*, *Fzd9*, and *Lef1* (*13*, *32*, *39*). To assess the impact of Wnt signaling pathway on RSC renewal, we first examined the effects of varying concentrations of iCRT 14, a Wnt inhibitor (*40*), and the absence of *fzd9b* on RSCs. Our findings revealed that in *fzd9b*^−/−^, cell aggregate differentiation, and proliferation were significantly reduced (*32*), accompanied by a substantial decrease in the number of renewing RSCs (Fig. 7, A and B). Western blot analysis revealed that Eya2 expression was markedly suppressed (Fig. 7C). Treatment with high concentrations of iCRT 14 (80 μM) effectively inhibited Wnt signaling, halting the renewal of RSCs and the reexpression of Eya2 (Fig. 7, D to F). In contrast, low concentrations of iCRT 14 (5 μM) enhanced RSC renewal (Fig. 7, D to F) but reduced expression of nephron differentiation markers *wnt4a* and *slc20a1a* (proximal tubule marker) (Fig. 7H) (*11*). Conversely, 6-bromoindirubin-3′-oxime (BIO) (10 μM), a Wnt activator (*41*), decreased RSC renewal (Fig. 7, D to E and G) while increasing expression of these markers (Fig. 7H). This indicates the necessity for balanced Wnt signaling in zebrafish RSC renewal.

Multiple studies have demonstrated that Wnt signaling plays a central role in balancing mammalian RPC renewal and differentiation (*42*–*45*). Elevated Wnt signaling can suppress Six2 function by interacting with β-catenin, thus favoring the expression of differentiation genes such as *Wnt4* and *Fgf8* in RPCs. Conversely, low Wnt signaling allows Six2 to associate with Tcf proteins, thereby inhibiting *Wnt4* and *Fgf8* expression and promoting mammalian RPC renewal (*42*). Given the extensive research on these processes in mammals, we now turn our attention to an unresolved question in zebrafish RSCs: Why does complete inhibition of Wnt activity suppress Eya2 expression and hinder RSC renewal?

We hypothesized that the expression of *eya2* is directly regulated by Wnt signaling. To test this, we engineered a luciferase reporter vector under the control of a 7-kb promoter region upstream of the *eya2* transcription start site. Lef1, a key nuclear factor in the Wnt pathway, interacts with β-catenin to regulate the expression of various target genes (*46*). Luciferase assays in HEK293T cells revealed that Lef1 overexpression significantly enhanced the *eya2* promoter activity (Fig. 7I). This activation was markedly reduced by the Wnt inhibitor iCRT 14 (40 μM), whereas the Wnt activator BIO (0.6 μM) increased promoter activity (Fig. 7I). These findings directly link Wnt signaling to the regulation of *eya2* expression. Furthermore, using the PROMO algorithm (*47*), we identified a Lef1 binding site within the *eya2* promoter at position −3923 to −3915 bp: CATCAAAG. To validate this prediction in vivo, we injected FLAG-tagged *lef1* mRNA into zebrafish embryos and subsequently used chromatin immunoprecipitation followed by polymerase chain reaction (ChIP-PCR). Our results confirmed that Lef1 specifically binds to the genomic region encompassing this predicted site in zebrafish (Fig. 7J). We also confirmed that Lef1 directly bound to this site using an electrophoretic mobility shift assay (EMSA) (Fig. 7K). Deletion of this site eliminated Lef1 binding (Fig. 7K) and abolished *eya2* promoter activity, rendering it unresponsive to modulation by either Lef1 overexpression or BIO treatment (Fig. 7L). Thus, Wnt signaling influenced the renewal of RSCs by directly regulating the reactivation of *eya2*. Therefore, Wnt signaling must achieve a precise equilibrium to facilitate nephron development and sustain the renewal of RSCs. Moreover, Wnt signaling plays a pivotal role in the activation of RSC renewal by directly regulating *eya2* expression.

## DISCUSSION

**Fig. 8.**
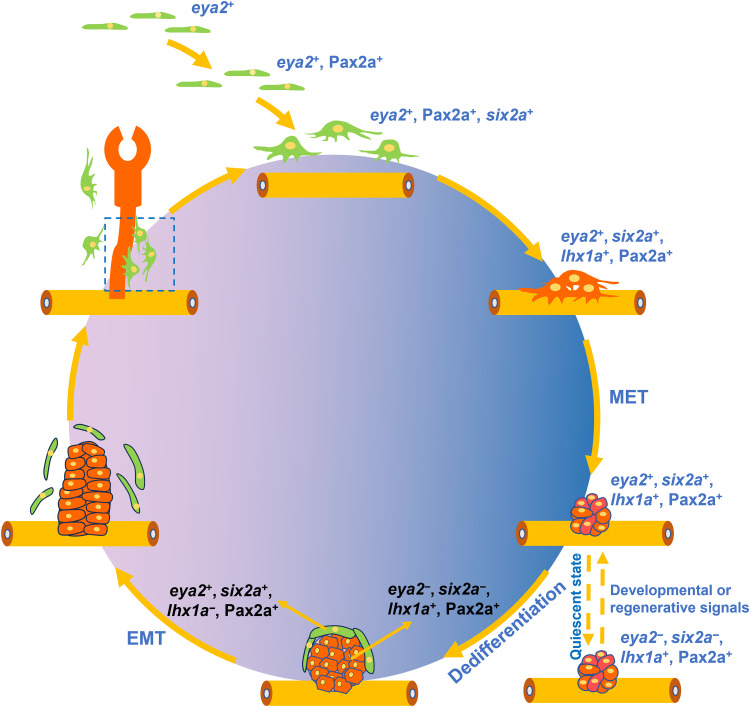
Schematic overview of zebrafish RSC renewal and differentiation. During zebrafish embryonic development, RSCs arise from *eya2*^+^ mesenchymal cells (shown as green cells). These cells then express Pax2a and *six2a*, maturing into functional RSCs. By 8 dpf, they begin expressing *lhx1a* (indicated as red cells) and aggregate, undergoing a MET to form compact cell aggregates. In the absence of developmental or injury-related signals, these aggregates enter a quiescent state. Upon reappearance of such signals, the aggregates reactivate: Base cells differentiate into nephrons, while apex cells regenerate RSCs through dedifferentiation and EMT. The newly formed RSCs reenter a cycle of generating new nephrons and additional RSCs.

Our study revealed that zebrafish RSCs arise from *eya2*^+^ mesenchymal cells during embryonic development. These cells sequentially activate Pax2a and *six2a*, leading to RSC maturation. By 8 dpf, RSCs expressed *lhx1a* and formed aggregates. These aggregates then undergo MET and rapidly proliferate. Aggregates basal cells differentiate into mature nephrons, whereas a subset of apex cells dedifferentiates, undergoes EMT, and detaches to form nascent RSCs. In adult kidneys, RSCs remain quiescent within the aggregates. Triggered by developmental or regenerative signals, these cells engage in differentiation and renewal processes, producing nascent nephrons and RSCs (Fig. 8). This previously unidentified cell type, characterized by quintessential stem cell properties such as sustained renewal capacity, multipotent differentiation potential, and the ability to undergo quiescence, was definitively identified as RSCs. Zebrafish RSCs uniquely add nephrons to existing nephrons, unlike mammalian nephrogenesis, which requires ureteric bud and RPC interactions (*9*, *15*). Since human adult kidneys lack ureteric buds, the zebrafish model offers a promising strategy. Studying zebrafish RSCs will provide essential insights for the development of human RSCs and advance nephron regeneration therapies.

In zebrafish, RSCs express *eya2*, which differs from the continuous expression of *Eya1* observed in mammalian RPCs. Both Eya1 and Eya2 are members of the Eya family and are characterized by a conserved EYA domain at the C terminus and lower conservation at the N terminus (*28*). Our study indicates that the genetic compensation of *eya1* in *eya2*^−/−^ cannot fully rescue RSC renewal, highlighting the stronger role of *eya2* in promoting RSC renewal. However, questions remain: Why do zebrafish RSCs use *eya*2, while mammals use *eya1*? What are the evolutionary reasons for and mechanisms underlying these differential gene expression patterns? These questions require further investigation.

Classical stem cells, such as hematopoietic stem cells, are characterized by their self-renewal ability, either through symmetric or asymmetric divisions, to maintain stemness in at least one daughter cell (*31*). Mammalian RPCs exhibit self-renewal for expansion but stop renewing around birth; hence, they are called progenitor cells (*3*, *4*). In organs such as the heart and liver, mature cells like cardiomyocytes and cholangiocytes can regenerate new cardiomyocytes and hepatocytes through dedifferentiation, proliferation, and redifferentiation (*48*–*50*). In contrast, zebrafish RSCs follow a distinct pattern: They first form aggregates and then transition into an epithelial-like intermediate state with high proliferative capacity. Aggregate apex cells, which reexpress *eya2* and *six2a* markers, dedifferentiate and undergo EMT to produce nascent RSCs. This cycle of differentiation, proliferation, and dedifferentiation characterizes RSC renewal in zebrafish. Consequently, we identified a previously unrecognized stem cell renewal mode that is distinct from the classical model. In addition, *eya2* is essential for this process. Yet, the transition from intermediate cells back to RSCs likely involves additional factors whose identification is crucial for the generation of human RSCs. Further investigation of these factors is essential. Simultaneously, quiescent RSC cell aggregates have been found in uninjured adult zebrafish kidneys, potentially enabling a swift kidney response to injury and improving survival in severe AKI. Mechanisms maintaining RSC quiescence are key topics for future research.

Balanced Wnt signaling is crucial for zebrafish RSC renewal and differentiation. Low Wnt activity promotes RSC renewal but decreases nephron differentiation, whereas high activity promotes the opposite. Zebrafish RSCs undergo gradient amplification during renewal. Our data showed that a cell aggregate formed by three to four RSCs could produce five to seven new RSCs, allowing for the progressive generation of more RSCs and nephrons. This mechanism ensures the simultaneous production of a significant number of nephrons during kidney development and regeneration. However, altering Wnt levels can disrupt this amplification process, leading to a shortage of nephrons or a continuous decrease in RSCs. Therefore, precise Wnt signaling is essential. In addition, our research indicates that Wnt signaling directly controls *eya2* expression, with Eya2 interacting with Six1b to activate critical RSC developmental genes. These mechanisms underscore the crucial role of Wnt signaling via Eya2 in both the differentiation and renewal of RSCs.

In summary, our study identified RSCs in zebrafish, with the main molecular markers determined as *eya2*, *six2a*, and Pax2a. RSCs undergo renewal through a differentiation-proliferation-dedifferentiation mode, which is distinct from classical stem cell self-renewal. Simultaneously, it was elucidated that the Wnt signaling pathway regulates the continuous renewal of RSCs through *eya2*. Unraveling the generation and renewal mechanisms of zebrafish RSCs will lay the foundation for developing therapeutic human RSCs.

## MATERIALS AND METHODS

### Zebrafish husbandry and transgenic lines

The zebrafish were bred, raised, and maintained according to standard protocols (*51*). The following transgenic lines were used: *Tg(cdh17:DsRed)* (*12*, *13*), *Tg(lhx1a:DsRed)* (*12*, *13*), *Tg(UAS:eGFP)* (*21*), *Tg(UAS:Kaede)* (*29*), *TgKI(six2a:p2a-Gal4-VP16)*, *Tg(pax2a:DsRed)*, *TgKI(eya1:p2a-Gal4-VP16)*, *TgKI(eya2:p2a-Gal4-VP16)*, *TgKI(osr1:p2a-Gal4-VP16)*, *TgKI(lhx1a:p2a-Gal4-VP16)*, *eya2*^−/−^, and *fzd9b*^−/−^. The AB strain was used as a WT control. For experiments involving adult zebrafish, individuals aged 6 to 12 months were selected, and approximately equal sex ratios were used. Anesthesia was achieved using 0.0168% buffered tricaine methanesulfonate (MS-222, Sigma-Aldrich). The animal care and use protocol was sanctioned by the Institutional Animal Care and Use Committee of the Army Medical University, China (approval number SYXK-PLA-2007035). Animal Research: Reporting of In Vivo Experiments (ARRIVE) guidelines have been followed where possible.

### Zebrafish model of AKI

AKI was induced in adult zebrafish by intraperitoneal injection of Gent as previously described (*11*, *14*). Specifically, a solution of Gent (2.7 μg/μl) was prepared, with each fish receiving a 20-μl dose via intraperitoneal administration. This procedure was performed on both the WT and other lines. Following the injection, each zebrafish was housed in an individual containment unit. The selection for further experimental analyses was based on the presence of proteinuria at 1 dpi.

### Single-cell RNA sequencing

Kidneys from 12 zebrafish, randomly selected at 4 dpi, were digested with 0.25% trypsin in 1.5-ml Eppendorf tubes to obtain single cells. These single cells were then subjected to a single-cell 3′ whole transcriptome amplification protocol. The libraries were sequenced on the Illumina NovaSeq platform, producing 150-bp paired-end reads at Novogene Bioinformatics Technology Co. Ltd., Tianjin, China. Quality assessment of the raw scRNA-seq reads was conducted using Fastp, which provides basic statistics to ensure data integrity. The scRNA-seq dataset comprised Read1 (R1), Read2 (R2), and i7 index read. The minimum read lengths were 60 bp for R1 and 42 bp for R2. R1 was essential for providing cell labels and molecular identifier information, whereas R2 provided crucial gene-specific details. Both R1 and R2 met quality standards for subsequent analysis. Data processing was conducted using UMI-tools (*52*) with the default parameters. The analytical pipeline included identifying authentic cells, extracting barcodes, filtering reads, mapping reads to the reference genome, assigning reads to specific genes, counting molecules, and generating gene-cell matrices. Data normalization, dimensionality reduction, clustering, and differential expression analyses were performed using the Seurat package (version 4.2). For clustering, we selected highly variable genes, and principal components derived from these genes were used to construct a graph segmented at a resolution of 0.6. Subclustering of the RSC population was accomplished by creating a new Seurat object containing only cells from cluster 15. The analytical steps of scaling, identification of highly variable genes, principal components analysis, and clustering were repeated, resulting in the identification of two distinct subclusters (sub 1 and sub 2). The sequencing data were archived in the Gene Expression Omnibus database under the accession code GSE 268624 (secure token: kxircimmblkxnat).

### CRISPR-Cas9 mutagenesis

The CRISPR-Cas9 target site within exon 2 of the *eya2* gene was identified using the CRISPR Design Tool (https://crisprscan.org/) (*53*). The guide RNA (gRNA) was designed to target the sequence 5′-GGGGGCGTCCCGCTGAAGTTTGG-3′ with the target sequence underlined. The gRNA construct included the T7 promoter and gRNA scaffold, which were cloned into the pMD19-T vector. The DNA template for gRNA was prepared by PCR amplification and then transcribed using the HiScribe T7 High Yield RNA Synthesis Kit (E2040S, New England Biolabs). For the mutagenesis, a mixture containing 1 nl of Cas9 protein (0.7 μM, M0646T, New England Biolabs) and gRNA (75 ng/μl) was injected into one-cell stage WT zebrafish embryos. Genomic DNA was extracted from the embryos at 24 hpf to assess the mutagenesis efficiency at the target site using PCR with primers listed in table S4. Sanger sequencing of the PCR products confirmed mutagenesis. Embryos exhibiting successful mutagenesis were grown until adulthood (F0 generation). The F1 generation was produced by mating F0 individuals with WT zebrafish, and genomic DNA from the tail tissue of F1 offspring was analyzed as described previously (*12*). The mutant allele *eya2*^+1^ was identified, validated using PCR and sequencing, and selected for further propagation. Thus, we established a stable *eya2* mutant line for further experimental analyses.

### Construction of transgenic lines

CRISPR-Cas9 knock-in was conducted as previously described (*21*). Briefly, a mixed solution containing 1 nl of Cas9 protein (0.7 μM, M0646T, New England Biolabs), gRNA (75 ng/μl), and donor plasmid harboring the knock-in sequence (30 ng/μl) was injected into one-cell stage WT embryos. Genomic DNA was extracted from these embryos at 24 hpf to verify the knock-in. Upon confirmation of knock-in, the treated embryos were raised to adulthood, similar to the F0 generation. Subsequent breeding of F0 individuals with WT zebrafish resulted in the F1 generation, which was also raised to adulthood. Genomic DNA was extracted from the tail tissues of F1 zebrafish and analyzed using PCR and sequencing. These confirmed that alleles were selectively propagated to establish a stable knock-in line for further analyses. The gRNA-targeted sequences, arm sequences, and primers used to verify the knock-in alleles are detailed in table S4. The knock-in zebrafish were hybridized to *Tg(UAS:eGFP)* lines to assess eGFP expression. This was performed to confirm whether the expression location of eGFP was consistent with previously reported patterns. To generate the *Tg(pax2a:DsRed)* construct, we first modified the previously established *lhx1a:DsRed/pI-SceI* plasmid (*12*, *13*) by replacing the *lhx1a* promoter with a 12-kb genomic fragment upstream of the *pax2a* transcription start site. The resulting *pax2a:DsRed/pI-SceI* plasmid was coinjected with I-SceI meganuclease (New England Biolabs, R0694S) into one-cell-stage zebrafish embryos. Injected embryos were raised to adulthood and screened for DsRed fluorescence in known *pax2a* expression tissues. A stable transgenic line, *Tg(pax2a:DsRed)*, was established on the basis of consistent germline transmission and tissue-specific expression.

### RSC serial transplantation

For the transplantation of *six2a:eGFP*^+^/*cdh17:DsRed*^−^/*lhx1a:DsRed*^−^ RSCs, adult AB zebrafish were used as recipients. The recipients were intraperitoneally injected with Gent to induce AKI. Four hours post-Gent injection, the fish underwent sublethal γ-irradiation at a dose of 30 Gy to prevent graft rejection (*11*). The donor zebrafish were *Tg(six2a:eGFP;cdh17:DsRed;lhx1a:DsRed)*. Kidneys dissected from these zebrafish at 4 dpi were treated with a 0.5% trypsin, 0.002% EDTA, and phosphate-buffered saline (PBS) for 2 min. They were then mechanically disrupted and filtered through a 40-μm nylon mesh. The eGFP^+^/DsRed^−^ cells were manually isolated using a mouth pipette and transferred onto a glass slide containing a drop of 1× PBS with 2% fetal calf serum. Cells were serially passed through three droplets of PBS/fetal calf serum to ensure the purity of nonpositive cells immediately before transplantation. Recipient fish were anesthetized using previously reported methods (*11*), and their kidneys were surgically exposed. Approximately 50 RSCs were injected into the renal trunk region of recipient zebrafish kidneys using a Hamilton syringe equipped with a 33-gauge needle, followed by surgical suturing. At 21 days posttransplantation, the recipient kidneys were excised to evaluate renewal and differentiation of the transplanted RSCs. The recipient kidneys containing RSCs were collected for subsequent transplantation.

### Photoconversion-based lineage tracing

At 16 dpf, *Tg(six2a:Kaede)* zebrafish larvae were immobilized in 1.0% low–melting point agarose for imaging procedures. Initial imaging was performed using dual 488/543-nm excitation wavelengths. Using the capability of the confocal microscope for targeted ultraviolet bleaching, a specific region of interest containing green Kaede–marked RSC aggregate was selected for photoconversion to red Kaede. After photoconversion, zebrafish were rescanned using the initial dual excitation wavelengths to confirm the shift from green to red fluorescence. The zebrafish were then released from the agarose and allowed to recover in the system water under dark conditions. Only those zebrafish that remained healthy postrecovery were selected for further analyses. Subsequently, the development of photoconverted cell aggregates was tracked every 2 to 4 days. The photoconverted zebrafish larvae were reimmobilized in 1.0% low–melting point agarose. Subsequently, the photoconverted regions were identified using their red fluorescence and reimaged. All photoconversion and imaging procedures were performed using a Nikon A1 confocal microscope.

### Preparation of anti-zebrafish podocin and Eya2 antibodies

We prepared anti-podocin, and anti-Eya2 antibodies were prepared as previously reported (*54*). Briefly, peptides corresponding to the C-terminal sequence of zebrafish podocin (CHGSDDGTKDSPM) and Eya2 (272 to 454 amino acids) were designed and synthesized. These peptides were then conjugated to keyhole limpet hemocyanin to generate immunogens. New Zealand white rabbits were immunized with these immunogens through a series of inoculations and blood collections, resulting in serum containing the antibodies. Last, specific anti-podocin and anti-Eya2 polyclonal antibodies were purified from the antiserum using protein A affinity chromatography, and their specificity was confirmed using Western blotting or immunofluorescence assays.

### Combined FISH and immunofluorescence

Combined FISH and immunofluorescence assays were performed as previously described (*55*). Briefly, kidneys were first fixed in 4% paraformaldehyde overnight at 4°C and then sectioned into 100-μm-thick slices. Sections underwent permeabilization with proteinase K (10 μg/ml), 0.1% Tween 20, and PBS for 20 min with gentle rocking. Riboprobes labeled with digoxigenin, corresponding to the zebrafish *eya2*, *six2a*, *wnt4a*, *lef1* (*13*), *eya1*, *six1b*, and *pax8* genes were synthesized from cDNA (table S4). These probes were detected using an anti–digoxigenin-peroxidase antibody (11207733910, Roche) combined with either the TSA Plus Cy3 (NEL744001KT, PerkinElmer) or TSA Plus Fluorescein systems (NEL741001KT, PerkinElmer). Following FISH, sections were processed for immunofluorescence staining. The primary antibodies used were anti-Pax2a (ab229318, Abcam) or anti-GFP (ab6658, Abcam). Detection was achieved using goat anti-rabbit immunoglobulin G (IgG) H&L conjugated with Alexa Fluor 633 (A21070, Thermo Fisher Scientific) or donkey anti-goat lgG conjugated with Alexa Fluor 647 (A-31573, Thermo Fisher Scientific). Confocal images were captured using a Nikon A1 microscope. The immunofluorescence assays for Pax2a and podocin described in the manuscript also used the aforementioned methods. Complete details of the antibodies used are listed in table S3.

### EdU assay

Cell proliferation in the zebrafish kidneys was evaluated using the Click-iT Plus EdU Alexa Fluor 647 Imaging Kit (C10640, Invitrogen). For juvenile zebrafish, 200 nl of 2 mM solution of EdU was injected into the heart. Three hours after injection, the juveniles were harvested and stained according to the manufacturer’s instructions provided with the kit. In adult zebrafish with uninjured kidneys, 20 μl of 200 μM EdU solution was administered intraperitoneally at 3-day intervals, with a total of seven injections. Subsequently, the kidneys were collected and stained to determine whether the *lhx1a*^+^ RSC aggregates were quiescent.

### Quantitative reverse transcription polymerase chain reaction

RNA was extracted from zebrafish kidney tissues using TRIzol reagent (15596018, Invitrogen). The cDNA was synthesized from the extracted RNA using the Prime Script II 1st Strand cDNA Synthesis Kit (9767, Takara). Quantitative PCR was then performed using TB Green Premix EX Taq II (RR820A, Takara). The primers used for amplifying *slc20a1a*, *wnt4a*, *eya1*, *eya2*, *six1b*, and *rpl13a* (*13*) are detailed in table S4. Gene expression levels were normalized to *rpl13a* mRNA expression to account for variations in input RNA.

### Coimmunoprecipitation

Recombinant proteins 3HA-Eya2 and 3Flag-Six1b were analyzed via coimmunoprecipitation using HA and Flag antibodies, respectively. HEK293T cells were cotransfected with *pCDNA3.1-3HA-eya2* and *pCDNA3.1-3Flag-six1b* vectors using Lipo8000 transfection reagent (C0533, Beyotime). Thirty-six hours posttransfection, cells were lysed using radioimmunoprecipitation assay buffer (P0013D, Beyotime) on ice. Lysates were incubated overnight with the respective antibodies at 4°C, followed by a 4-hour incubation with protein A/G-coupled beads at 4°C. The beads were then washed three times with washing buffer [50 mM tris-HCl (pH 7.4), 150 mM NaCl, and 0.5% Triton X-100]. Proteins were eluted using 100 mM glycine-HCl buffer (pH 3.0) and analyzed by Western blotting. Detailed information on the antibodies used is provided in table S3.

### Western blotting

Zebrafish kidneys were homogenized using a 1-ml syringe and a needle in cell lysis buffer [50 mM PBS (pH 7.4), 1% SDS, and 0.5% Triton X-100] supplemented with protease inhibitor (P1005, Beyotime). The homogenate was centrifuged at 12,000*g* for 20 min at 4°C, and the supernatant was collected as the protein sample. Protein concentration was quantified using a bicinchoninic acid protein assay kit (BL521A, Biosharp). Western blotting was performed according to standard procedures. The protein levels of Eya2 and β-actin were detected using enhanced chemiluminescence (ECL) with specific antibodies, as detailed in table S3. A rabbit polyclonal antibody against zebrafish Eya2, developed by HUABIO, was used.

### Pharmaceutical treatment

During kidney regeneration, iCRT 14 (5 or 80 μM, 10 μl per fish), BIO (10 μM, 10 μl per fish), or 0.1% dimethyl sulfoxide (10 μl per fish) was intraperitoneally injected individually every other day starting from 2 dpi until the kidneys were collected for further analysis. Detailed information about these inhibitors is listed in table S3.

### Vivo-morpholino

The vivo-MOs were designed as previously described and are listed in table S4 (*56*–*58*). During kidney regeneration, the *eya2* vivo-MO (40 μm, 20 μl), *six1b* vivo-MO (40 μm, 20 μl), and control vivo-MO (40 μm, 20 μl) were intraperitoneally injected at −1, 2, and 4 dpi. The kidneys were collected at 5 dpi for further analysis. Detailed vivo-MO information is listed in table S4.

### Luciferase assay

Reporter plasmids harboring WT and mutant (Δ) sequences of the 7-kb *eya2* promoter, as detailed in table S4, were constructed. These plasmids and *pCDNA3.1-3HA-lef1* were cotransfected into HEK293T cells using Lipo8000 (C0533, Beyotime). Twelve hours posttransfection, cells were treated with either iCRT 14 (40 μM) or BIO (0.6 μM). Luciferase activity was assessed 36 hours after transfection using the Luciferase Assay Kit (RG044S, Beyotime).

### ChIP-PCR assay

ChIP assay was performed using the ChIP assay kit (Beyotime, P2080S) according to the user manual. Briefly, 3Flag-lef1 mRNA was synthesized using SP6 kit (Thermo Fisher Scientific, AM1340) and injected into one-cell stage WT zebrafish embryos. The cells of 200 injected embryos were collected at 72 hpf and incubated with formaldehyde (1%) for 2 hours at 29°C to cross-link the nuclear proteins to DNA. Subsequently, cells were washed with ice-cold PBS and lysed in SDS lysis buffer followed by sonication and immunoprecipitation with anti-Flag antibody (Cell Signaling Technology, 14793) or rabbit IgG (Beyotime, A7016). The captured chromatin was eluted and uncross-linked, and the DNA was recovered. The ChIP-isolated DNA was subjected to PCR analyses using the primer pair spanning *eya2* promoter. The primer sequences were listed in table S4.

### Electrophoretic mobility shift assay

The direct binding of Lef1 to the *eya2* promoter sequence 5′-CATCAAAG-3′ was examined using an EMSA, following established protocols. Recombinant 3Flag-Lef1 proteins were expressed in HEK293T cells and purified using Anti-DYKDDDDK Affinity Beads (SA042001, Smart-Life Sciences). Both WT and mutant (Δ) probes, synthesized with 5′ digoxigenin-labeled primers (table S4), were incubated with 3Flag-Lef1 in binding buffer [100 mM Hepes (pH 7.5), 100 mM NaCl, 50 mM KCl, 1 mM dithiothreitol, 1 mM EDTA, and 5% glycerol] for 30 min at 25°C. The resulting complexes were resolved on a tris-borate EDTA–polyacrylamide gel electrophoresis and subsequently transferred onto a nylon membrane. Probe detection was using an anti–digoxigenin-peroxidase antibody (11207733910, Roche) and ECL.

### Statistics

Unless otherwise specified, all experiments were conducted with a minimum of three independent replicates. Results are presented as means ± SDs. Statistical analyses were performed using Microsoft Excel (Office Home and Student 2019 version) and GraphPad Prism (version 8.02) for Windows. Data were subjected to two-sided *t* tests and are reported as mean values ± SDs.

## References

[1] P. Romagnani, G. Remuzzi, R. Glassock, A. Levin, K. J. Jager, M. Tonelli, Z. Massy, C. Wanner, H. J. Anders, Chronic kidney disease. Nat. Rev. Dis. Primers. 3, 17088 (2017). 29168475 10.1038/nrdp.2017.88

[2] J. F. Bertram, R. N. Douglas-Denton, B. Diouf, M. D. Hughson, W. E. Hoy, Human nephron number: Implications for health and disease. Pediatr. Nephrol. 26, 1529–1533 (2011). 21604189 10.1007/s00467-011-1843-8

[3] H. A. Hartman, H. L. Lai, L. T. Patterson, Cessation of renal morphogenesis in mice. Dev. Biol. 310, 379–387 (2007). 17826763 10.1016/j.ydbio.2007.08.021 PMC2075093

[4] B. A. Rumballe, K. M. Georgas, A. N. Combes, A. L. Ju, T. Gilbert, M. H. Little, Nephron formation adopts a novel spatial topology at cessation of nephrogenesis. Dev. Biol. 360, 110–122 (2011). 21963425 10.1016/j.ydbio.2011.09.011 PMC6186757

[5] Y. Rinkevich, D. T. Montoro, H. Contreras-Trujillo, O. Harari-Steinberg, A. M. Newman, J. M. Tsai, X. Lim, R. Van-Amerongen, A. Bowman, M. Januszyk, O. Pleniceanu, R. Nusse, M. T. Longaker, I. L. Weissman, B. Dekel, In vivo clonal analysis reveals lineage-restricted progenitor characteristics in mammalian kidney development, maintenance, and regeneration. Cell Rep. 7, 1270–1283 (2014). 24835991 10.1016/j.celrep.2014.04.018 PMC4425291

[6] R. Asao, T. Seki, M. Takagi, H. Yamada, F. Kodama, Y. Hosoe-Nagai, E. Tanaka, J. A. O. Trejo, K. Yamamoto-Nonaka, Y. Sasaki, T. Hidaka, T. Ueno, M. Yanagita, Y. Suzuki, Y. Tomino, K. Asanuma, Rac1 in podocytes promotes glomerular repair and limits the formation of sclerosis. Sci. Rep. 8, 5061 (2018). 29567961 10.1038/s41598-018-23278-6 PMC5864960

[7] A. Peired, E. Lazzeri, L. Lasagni, P. Romagnani, Glomerular regeneration: When can the kidney regenerate from injury and what turns failure into success? Nephron Exp. Nephrol. 126, 70–75 (2014). 24854644 10.1159/000360669

[8] G. F. Gerlach, R. A. Wingert, Kidney organogenesis in the zebrafish: Insights into vertebrate nephrogenesis and regeneration. Wiley Interdiscip. Rev. Dev. Biol. 2, 559–585 (2013). 24014448 10.1002/wdev.92 PMC3772547

[9] M. H. Little, A. P. McMahon, Mammalian kidney development: Principles, progress, and projections. Cold Spring Harb. Perspect. Biol. 4, a008300 (2012). 22550230 10.1101/cshperspect.a008300 PMC3331696

[10] R. Reimschuessel, A fish model of renal regeneration and development. ILAR J. 42, 285–291 (2001). 11581520 10.1093/ilar.42.4.285

[11] C. Q. Diep, D. Ma, R. C. Deo, T. M. Holm, R. W. Naylor, N. Arora, R. A. Wingert, F. Bollig, G. Djordjevic, B. Lichman, H. Zhu, T. Ikenaga, F. Ono, C. Englert, C. A. Cowan, N. A. Hukriede, R. I. Handin, A. J. Davidson, Identification of adult nephron progenitors capable of kidney regeneration in zebrafish. Nature 470, 95–100 (2011). 21270795 10.1038/nature09669 PMC3170921

[12] C. Liu, X. Liu, Z. He, J. Zhang, X. Tan, W. Yang, Y. Zhang, T. Yu, S. Liao, L. Dai, Z. Xu, F. Li, Y. Huang, J. Zhao, Proenkephalin-A secreted by renal proximal tubules functions as a brake in kidney regeneration. Nat. Commun. 14, 7167 (2023). 37935684 10.1038/s41467-023-42929-5 PMC10630464

[13] X. Liu, T. Yu, X. Tan, D. Jin, W. Yang, J. Zhang, L. Dai, Z. He, D. Li, Y. Zhang, S. Liao, J. Zhao, T. P. Zhong, C. Liu, Renal interstitial cells promote nephron regeneration by secreting prostaglandin E2. eLife 12, e81438 (2023). 36645741 10.7554/eLife.81438 PMC9943066

[14] J. Augusto, B. Smith, S. Smith, J. Robertson, R. Reimschuessel, Gentamicin-induced nephrotoxicity and nephroneogenesis in Oreochromis nilotica, a tilapian fish. Dis. Aquat. Organ. 26, 49–58 (1996).

[15] I. Pietila, S. J. Vainio, Kidney development: An overview. Nephron Exp. Nephrol. 126, 40–44 (2014). 24854638 10.1159/000360659

[16] M. Bouchard, A. Souabni, M. Mandler, A. Neubuser, M. Busslinger, Nephric lineage specification by Pax2 and Pax8. Genes Dev. 16, 2958–2970 (2002). 12435636 10.1101/gad.240102 PMC187478

[17] A. Kobayashi, M. T. Valerius, J. W. Mugford, T. J. Carroll, M. Self, G. Oliver, A. P. McMahon, Six2 defines and regulates a multipotent self-renewing nephron progenitor population throughout mammalian kidney development. Cell Stem Cell 3, 169–181 (2008). 18682239 10.1016/j.stem.2008.05.020 PMC2561900

[18] T. E. Tsang, W. Shawlot, S. J. Kinder, A. Kobayashi, K. M. Kwan, K. Schughart, A. Kania, T. M. Jessell, R. R. Behringer, P. P. Tam, Lim1 activity is required for intermediate mesoderm differentiation in the mouse embryo. Dev. Biol. 223, 77–90 (2000). 10864462 10.1006/dbio.2000.9733

[19] R. G. James, C. N. Kamei, Q. Wang, R. Jiang, T. M. Schultheiss, Odd-skipped related 1 is required for development of the metanephric kidney and regulates formation and differentiation of kidney precursor cells. Development 133, 2995–3004 (2006). 16790474 10.1242/dev.02442

[20] J. Li, C. Cheng, J. Xu, T. Zhang, B. Tokat, G. Dolios, A. Ramakrishnan, L. Shen, R. Wang, P. X. Xu, The transcriptional coactivator Eya1 exerts transcriptional repressive activity by interacting with REST corepressors and REST-binding sequences to maintain nephron progenitor identity. Nucleic Acids Res. 50, 10343–10359 (2022). 36130284 10.1093/nar/gkac760 PMC9561260

[21] J. Li, B. B. Zhang, Y. G. Ren, S. Y. Gu, Y. H. Xiang, J. L. Du, Intron targeting-mediated and endogenous gene integrity-maintaining knockin in zebrafish using the CRISPR/Cas9 system. Cell Res. 25, 634–637 (2015). 25849248 10.1038/cr.2015.43 PMC4423083

[22] M. E. Halpern, J. Rhee, M. G. Goll, C. M. Akitake, M. Parsons, S. D. Leach, Gal4/UAS transgenic tools and their application to zebrafish. Zebrafish 5, 97–110 (2008). 18554173 10.1089/zeb.2008.0530 PMC6469517

[23] D. Grote, A. Souabni, M. Busslinger, M. Bouchard, Pax 2/8-regulated Gata 3 expression is necessary for morphogenesis and guidance of the nephric duct in the developing kidney. Development 133, 53–61 (2006). 16319112 10.1242/dev.02184

[24] Y. Atsuta, Y. Takahashi, FGF8 coordinates tissue elongation and cell epithelialization during early kidney tubulogenesis. Development 142, 2329–2337 (2015). 26130757 10.1242/dev.122408 PMC4510593

[25] T. Xie, Y. Wang, N. Deng, G. Huang, F. Taghavifar, Y. Geng, N. Liu, V. Kulur, C. Yao, P. Chen, Z. Liu, B. Stripp, J. Tang, J. Liang, P. W. Noble, D. Jiang, Single-cell deconvolution of fibroblast heterogeneity in mouse pulmonary fibrosis. Cell Rep. 22, 3625–3640 (2018). 29590628 10.1016/j.celrep.2018.03.010 PMC5908225

[26] T. Sudo, T. Iwaya, N. Nishida, G. Sawada, Y. Takahashi, M. Ishibashi, K. Shibata, H. Fujita, K. Shirouzu, M. Mori, K. Mimori, Expression of mesenchymal markers vimentin and fibronectin: The clinical significance in esophageal squamous cell carcinoma. Ann. Surg. Oncol. 20, 324–335 (2013). 10.1245/s10434-012-2418-z 22644514

[27] C. Vannier, K. Mock, T. Brabletz, W. Driever, Zeb1 regulates E-cadherin and Epcam (epithelial cell adhesion molecule) expression to control cell behavior in early zebrafish development. J. Biol. Chem. 288, 18643–18659 (2013). 23667256 10.1074/jbc.M113.467787 PMC3696638

[28] T. Ishihara, K. Ikeda, S. Sato, H. Yajima, K. Kawakami, Differential expression of Eya1 and Eya2 during chick early embryonic development. Gene Expr. Patterns 8, 357–367 (2008). 18316249 10.1016/j.gep.2008.01.003

[29] K. Hatta, H. Tsujii, T. Omura, Cell tracking using a photoconvertible fluorescent protein. Nat. Protoc. 1, 960–967 (2006). 17406330 10.1038/nprot.2006.96

[30] M. A. Saleem, L. Ni, I. Witherden, K. Tryggvason, V. Ruotsalainen, P. Mundel, P. W. Mathieson, Co-localization of nephrin, podocin, and the actin cytoskeleton: Evidence for a role in podocyte foot process formation. Am. J. Pathol. 161, 1459–1466 (2002). 12368218 10.1016/S0002-9440(10)64421-5 PMC1867300

[31] E. Fuchs, T. Chen, A matter of life and death: Self-renewal in stem cells. EMBO Rep. 14, 39–48 (2013). 23229591 10.1038/embor.2012.197 PMC3537149

[32] C. N. Kamei, T. F. Gallegos, Y. Liu, N. Hukriede, I. A. Drummond, Wnt signaling mediates new nephron formation during zebrafish kidney regeneration. Development 146, (2019). 10.1242/dev.168294 PMC6503981 31036548

[33] J. An, Y. Zheng, C. T. Dann, Mesenchymal to epithelial transition mediated by CDH1 promotes spontaneous reprogramming of male germline stem cells to pluripotency. Stem Cell Rep. 8, 446–459 (2017). 10.1016/j.stemcr.2016.12.006 PMC5311464 28065642

[34] S. Nakajima, R. Doi, E. Toyoda, S. Tsuji, M. Wada, M. Koizumi, S. S. Tulachan, D. Ito, K. Kami, T. Mori, Y. Kawaguchi, K. Fujimoto, R. Hosotani, M. Imamura, N-cadherin expression and epithelial-mesenchymal transition in pancreatic carcinoma. Clin. Cancer Res. 10, 4125–4133 (2004). 15217949 10.1158/1078-0432.CCR-0578-03

[35] A. de Morree, T. A. Rando, Regulation of adult stem cell quiescence and its functions in the maintenance of tissue integrity. Nat. Rev. Mol. Cell Biol. 24, 334–354 (2023). 36922629 10.1038/s41580-022-00568-6 PMC10725182

[36] A. Rossi, Z. Kontarakis, C. Gerri, H. Nolte, S. Holper, M. Kruger, D. Y. Stainier, Genetic compensation induced by deleterious mutations but not gene knockdowns. Nature 524, 230–233 (2015). 26168398 10.1038/nature14580

[37] A. N. Patrick, J. H. Cabrera, A. L. Smith, X. S. Chen, H. L. Ford, R. Zhao, Structure-function analyses of the human SIX1-EYA2 complex reveal insights into metastasis and BOR syndrome. Nat. Struct. Mol. Biol. 20, 447–453 (2013). 23435380 10.1038/nsmb.2505 PMC3618615

[38] P. X. Xu, W. Zheng, L. Huang, P. Maire, C. Laclef, D. Silvius, Six1 is required for the early organogenesis of mammalian kidney. Development 130, 3085–3094 (2003). 12783782 10.1242/dev.00536 PMC3872112

[39] D. M. Iglesias, P. A. Hueber, L. Chu, R. Campbell, A. M. Patenaude, A. J. Dziarmaga, J. Quinlan, O. Mohamed, D. Dufort, P. R. Goodyer, Canonical WNT signaling during kidney development. Am. J. Physiol. Renal Physiol. 293, F494–F500 (2007). 17494089 10.1152/ajprenal.00416.2006

[40] K. Spivack, C. Muzzelo, M. Hall, E. Warga, C. Neely, H. Slepian, A. Cunningham, M. Tucker, J. Elmer, Enhancement of transgene expression by the beta-catenin inhibitor iCRT14. Plasmid 114, 102556 (2021). 33472046 10.1016/j.plasmid.2021.102556

[41] X. Huang, L. Zhong, J. Hendriks, J. N. Post, M. Karperien, The effects of the WNT-signaling modulators BIO and PKF118-310 on the chondrogenic differentiation of human mesenchymal stem cells. Int. J. Mol. Sci. 19, 561 (2018). 29438298 10.3390/ijms19020561 PMC5855783

[42] J. S. Park, W. Ma, L. L. O’Brien, E. Chung, J. J. Guo, J. G. Cheng, M. T. Valerius, J. A. McMahon, W. H. Wong, A. P. McMahon, Six2 and Wnt regulate self-renewal and commitment of nephron progenitors through shared gene regulatory networks. Dev. Cell 23, 637–651 (2012). 22902740 10.1016/j.devcel.2012.07.008 PMC3892952

[43] C. M. Karner, A. Das, Z. Ma, M. Self, C. Chen, L. Lum, G. Oliver, T. J. Carroll, Canonical Wnt9b signaling balances progenitor cell expansion and differentiation during kidney development. Development 138, 1247–1257 (2011). 21350016 10.1242/dev.057646 PMC3050658

[44] H. Ramalingam, A. R. Fessler, A. Das, M. T. Valerius, J. Basta, L. Robbins, A. C. Brown, L. Oxburgh, A. P. McMahon, M. Rauchman, T. J. Carroll, Disparate levels of beta-catenin activity determine nephron progenitor cell fate. Dev. Biol. 440, 13–21 (2018). 29705331 10.1016/j.ydbio.2018.04.020 PMC5988999

[45] H. Bugacov, B. Der, B. M. Briantseva, Q. Guo, S. Kim, N. O. Lindstrom, A. P. McMahon, Dose-dependent responses to canonical Wnt transcriptional complexes in the regulation of mammalian nephron progenitors. Development 151, dev202279 (2024). 39250420 10.1242/dev.202279 PMC11463962

[46] Q. Eastman, R. Grosschedl, Regulation of LEF-1/TCF transcription factors by Wnt and other signals. Curr. Opin. Cell Biol. 11, 233–240 (1999). 10209158 10.1016/s0955-0674(99)80031-3

[47] X. Messeguer, R. Escudero, D. Farre, O. Nunez, J. Martinez, M. M. Alba, PROMO: Detection of known transcription regulatory elements using species-tailored searches. Bioinformatics 18, 333–334 (2002). 11847087 10.1093/bioinformatics/18.2.333

[48] A. Raven, W. Y. Lu, T. Y. Man, S. Ferreira-Gonzalez, E. O’Duibhir, B. J. Dwyer, J. P. Thomson, R. R. Meehan, R. Bogorad, V. Koteliansky, Y. Kotelevtsev, C. Ffrench-Constant, L. Boulter, S. J. Forbes, Cholangiocytes act as facultative liver stem cells during impaired hepatocyte regeneration. Nature 547, 350–354 (2017). 28700576 10.1038/nature23015 PMC5522613

[49] Y. Yao, C. Wang, Dedifferentiation: Inspiration for devising engineering strategies for regenerative medicine. NPJ Regen. Med. 5, 14 (2020). 32821434 10.1038/s41536-020-00099-8 PMC7395755

[50] C. Jopling, E. Sleep, M. Raya, M. Marti, A. Raya, J. C. Izpisua Belmonte, Zebrafish heart regeneration occurs by cardiomyocyte dedifferentiation and proliferation. Nature 464, 606–609 (2010). 20336145 10.1038/nature08899 PMC2846535

[51] M. Westerfield, *The Zebrafish Book: A Guide for the Laboratory Use of Zebrafish* (University of Oregon Press, Eugene 4th ed., 2000).

[52] T. Smith, A. Heger, I. Sudbery, UMI-tools: Modeling sequencing errors in Unique Molecular Identifiers to improve quantification accuracy. Genome Res. 27, 491–499 (2017). 28100584 10.1101/gr.209601.116 PMC5340976

[53] M. A. Moreno-Mateos, C. E. Vejnar, J. D. Beaudoin, J. P. Fernandez, E. K. Mis, M. K. Khokha, A. J. Giraldez, CRISPRscan: Designing highly efficient sgRNAs for CRISPR-Cas9 targeting in vivo. Nat. Methods 12, 982–988 (2015). 26322839 10.1038/nmeth.3543 PMC4589495

[54] L. Wang, L. Zhang, Q. Hou, X. Zhu, Z. Chen, Z. Liu, Triptolide attenuates proteinuria and podocyte apoptosis via inhibition of NF-κB/GADD45B. Sci. Rep. 8, 10843 (2018). 30022148 10.1038/s41598-018-29203-1 PMC6052061

[55] J. He, D. Mo, J. Chen, L. Luo, Combined whole-mount fluorescence in situ hybridization and antibody staining in zebrafish embryos and larvae. Nat. Protoc. 15, 3361–3379 (2020). 32908315 10.1038/s41596-020-0376-7

[56] C. Y. Lin, W. T. Chen, H. C. Lee, P. H. Yang, H. J. Yang, H. J. Tsai, The transcription factor Six1a plays an essential role in the craniofacial myogenesis of zebrafish. Dev. Biol. 331, 152–166 (2009). 19409884 10.1016/j.ydbio.2009.04.029

[57] J. Wang, Y. Wu, F. Zhao, Y. Wu, W. Dong, J. Zhao, Z. Zhu, D. Liu, Fgf-signaling-dependent Sox9a and Atoh1a regulate otic neural development in zebrafish. J. Neurosci. 35, 234–244 (2015). 25568117 10.1523/JNEUROSCI.3353-14.2015 PMC6605248

[58] J. Chen, T. Yu, X. He, Y. Fu, L. Dai, B. Wang, Y. Wu, J. He, Y. Li, F. Zhang, J. Zhao, C. Liu, Dual roles of hydrogen peroxide in promoting zebrafish renal repair and regeneration. Biochem. Biophys. Res. Commun. 516, 680–685 (2019). 31248596 10.1016/j.bbrc.2019.06.052

