# Supplementary material for: Identification of renal stem cells in zebrafish
Source: Sci Adv. 2025 Aug 22;11(34):eadx5296. doi: 10.1126/sciadv.adx5296 (PMC12372851; doi:10.1126/sciadv.adx5296)
Supplement: Supplementary file 1 — Figs. S1 to S10 Legends for movies S1 to S3 Legends for tables S1 and S2 Tables S3 and S4 [file sciadv.adx5296_sm.pdf]

Science Advances
AAAS

Supplementary Materials for

Identification of renal stem cells in zebrafish

Ting Yu *et al.*

Corresponding author: Chi Liu, chiliu@tmmu.edu.cn; Jinghong Zhao, zhaojh@tmmu.edu.cn

Sci. Adv. **11**, eadx5296 (2025)
DOI: 10.1126/sciadv.adx5296

The PDF file includes:

Figs. S1 to S10
Legends for movies S1 to S3
Legends for tables S1 and S2
Tables S3 and S4

Other Supplementary Material for this manuscript includes the following:

Movies S1 to S3
Tables S1 and S2

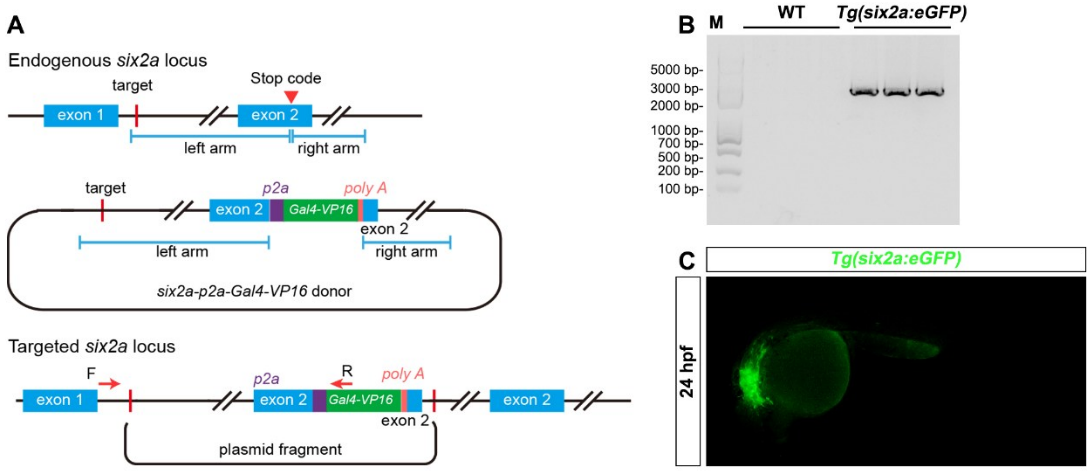

fig. S1. Intron targeting-mediated Gal4-VP16 knockin at the zebrafish *six2a* locus. (**A**) Schematic illustration of the intron targeting-mediated strategy for generating a *Gal4-VP16* knockin at the zebrafish *six2a* locus using the CRISPR/Cas9 system. (**B**) PCR analysis to verify the successful construction of the *TgKI(six2a:p2a-Gal4-VP16)* transgene in F1 zebrafish. The locations of the forward (F) and reverse (R) primers are indicated in panel A. (**C**) Representative projected in vivo confocal images of *Tg(six2a:eGFP)* larvae at 24 hpf.

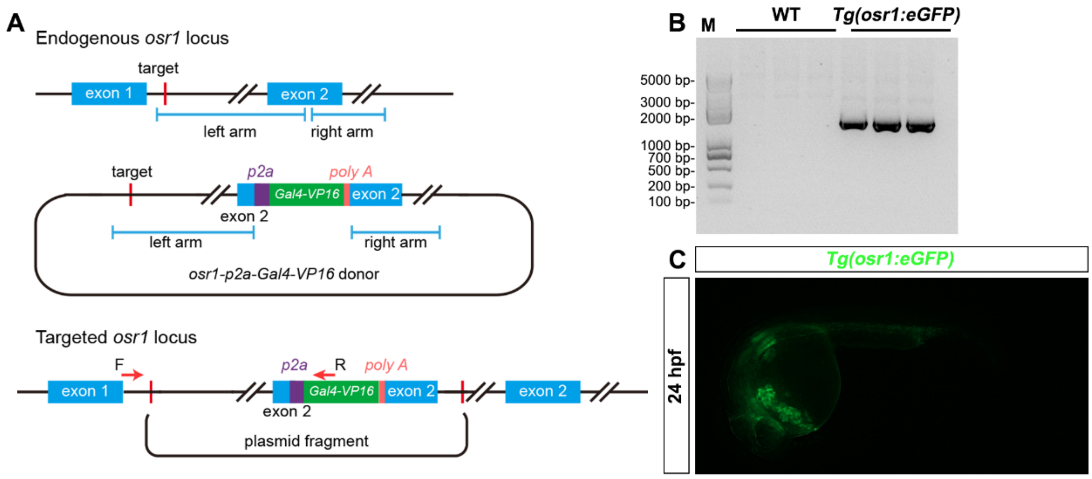

fig. S2. Intron targeting-mediated *Gal4-VP16* knockin at the zebrafish *osr1* locus. (**A**) Schematic illustration of the intron targeting-mediated strategy for generating a *Gal4-VP16* knockin at the zebrafish *osr1* locus using the CRISPR/Cas9 system. (**B**) PCR analysis to verify the successful construction of the *TgKI(osr1:p2a-Gal4-VP16)* transgene in F1 zebrafish. The locations of the forward (F) and reverse (R) primers are indicated in panel A. (**C**) Representative projected in vivo confocal images of *Tg(osr1:eGFP)* larvae at 24 hpf.

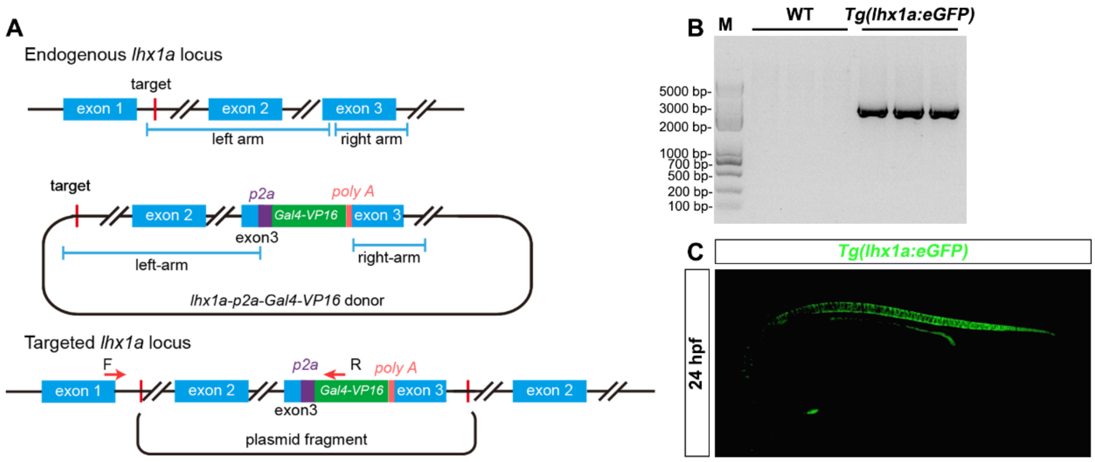

fig. S3. Intron targeting-mediated *Gal4-VP16* knockin at the zebrafish *lhx1a* locus. (**A**) Schematic illustration of the intron targeting-mediated strategy for generating a *Gal4-VP16* knockin at the zebrafish *lhx1a* locus using the CRISPR/Cas9 system. (**B**) PCR analysis to verify the successful construction of the *TgKI(lhx1a:p2a-Gal4-VP16)* transgene in F1 zebrafish. The locations of the forward (F) and reverse (R) primers are indicated in panel A. (**C**) Representative projected in vivo confocal images of *Tg(lhx1a:eGFP)* larvae at 24 hpf.

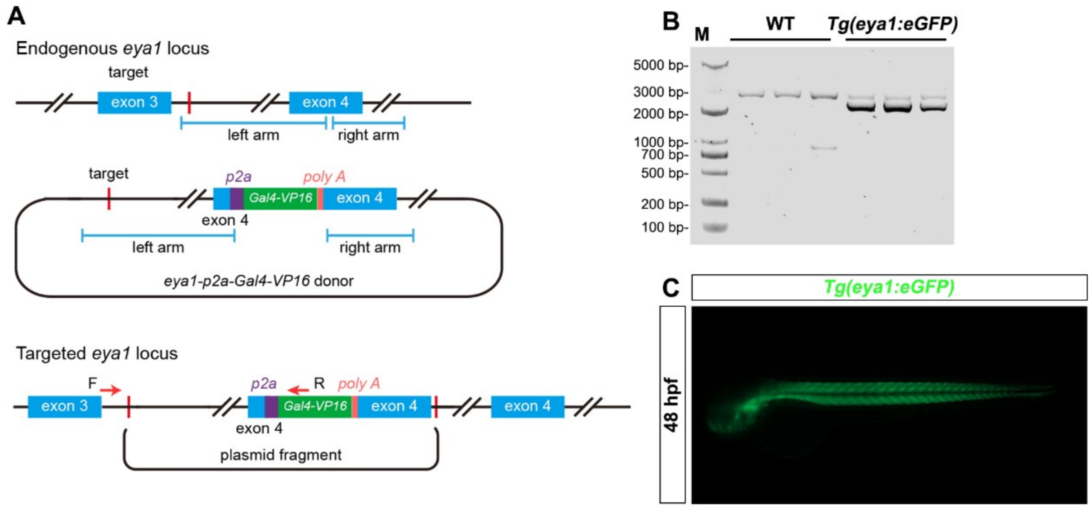

fig. S4. Intron targeting-mediated *Gal4-VP16* knockin at the zebrafish *eya1* locus. (**A**) Schematic illustration of the intron targeting-mediated strategy for generating a *Gal4-VP16* knockin at the zebrafish *eya1* locus using the CRISPR/Cas9 system. (**B**) PCR analysis to verify the successful construction of the *TgKI(eya1:p2a-Gal4-VP16)* transgene in F1 zebrafish. The locations of the forward (F) and reverse (R) primers are indicated in panel A. (**C**) Representative projected in vivo confocal images of *Tg(eya1:eGFP)* larvae at 24 hpf.

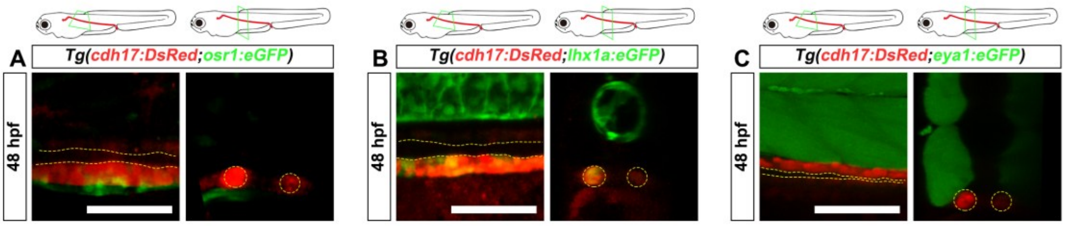

fig. S5. RSCs cannot be labeled by mammalian RPC markers in 48 hpf zebrafish. (**A–C**) Confocal images showing the regions of RSCs production in 48 hpf zebrafish: (A) *Tg(cdh17:DsRed;osr1:eGFP)*, (B) *Tg(cdh17:DsRed;lhx1a:eGFP)* and (C) *Tg(cdh17:DsRed;eya1:eGFP)*. The yellow dashed lines indicate the inner sides of the two pronephric tubules. Scale bars, 100 μm.

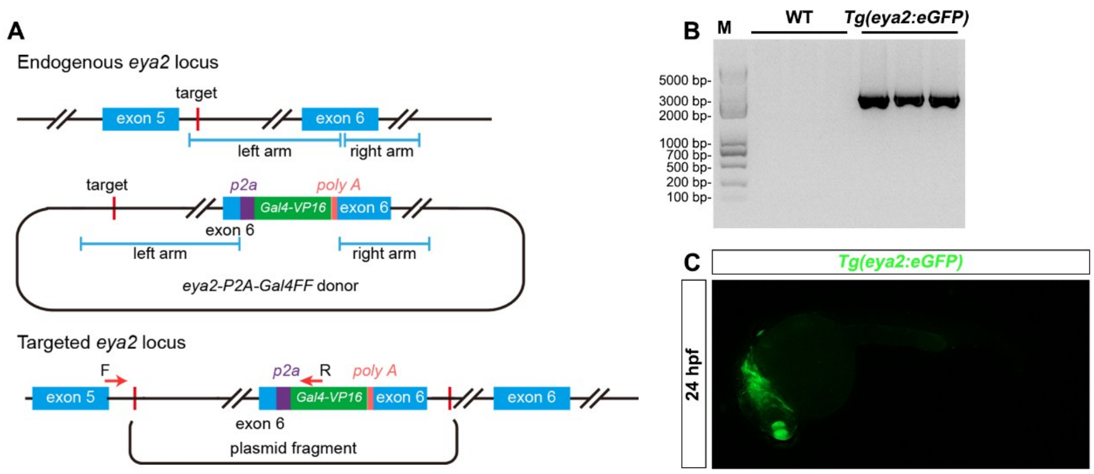

fig. S6. Intron targeting-mediated *Gal4-VP16* knockin at the zebrafish *eya2* locus. (**A**) Schematic illustration of the intron targeting-mediated strategy for generating a *Gal4-VP16* knockin at the zebrafish *eya2* locus using the CRISPR/Cas9 system. (**B**) PCR analysis to verify the successful construction of the *TgKI(eya2:p2a-Gal4-VP16)* transgene in F1 zebrafish. The locations of the forward (F) and reverse (R) primers are indicated in panel A. (**C**) Representative projected in vivo confocal images of *Tg(eya2:eGFP)* larvae at 24 hpf.

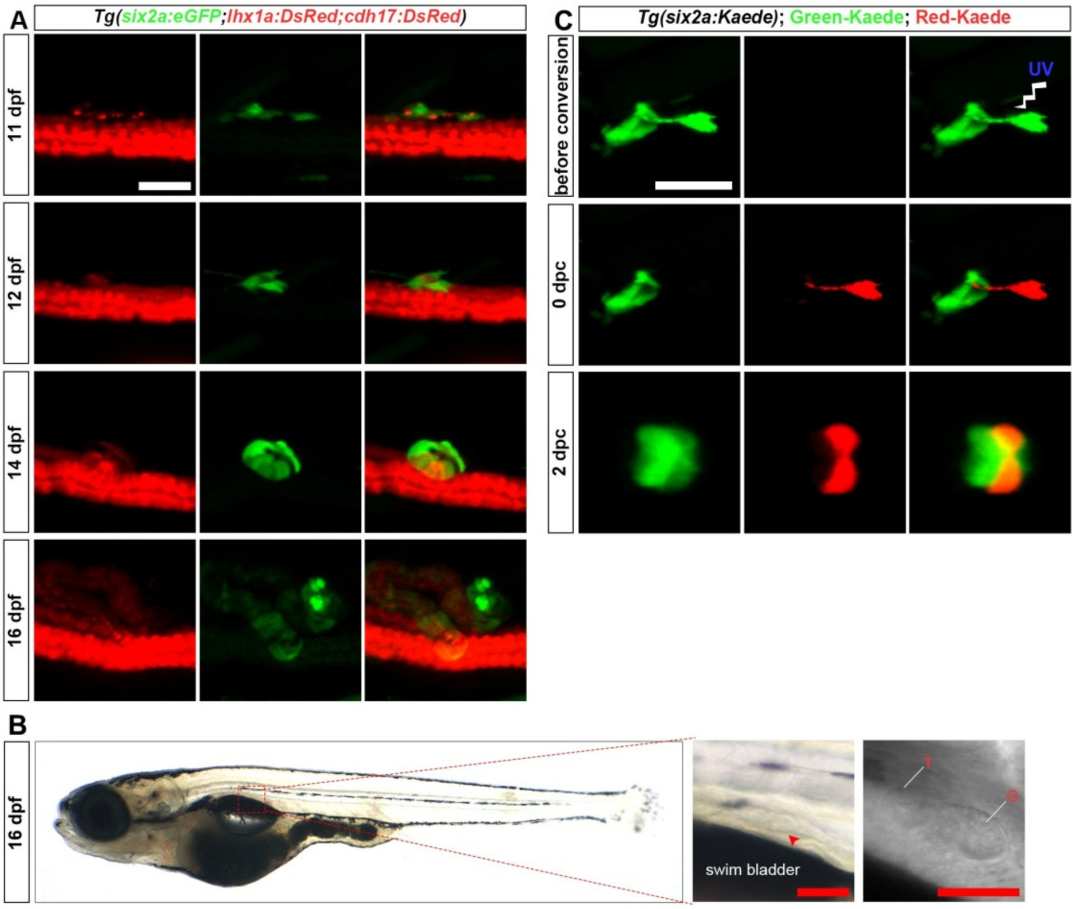

fig. S7. Differentiation of *six2a*⁺ RSCs into nephrons. (A) Confocal images showing time-lapse observations of *Tg(six2a:eGFP;lhx1a:DsRed;cdh17:DsRed)* zebrafish from 11 to 16 dpf. After forming cell aggregates, RSCs began to differentiate into RVs, which subsequently developed into nephrons. **(B)** Bright-field images clearly showing the newly formed nephron (arrowhead). G: glomerulus, T: tubule. **(C)** In the *Tg(six2a:kaede)* line, individual RSCs were photoconverted and subsequently tracked, revealing their ability to cluster with other RSCs to form cell aggregate. Scale bars in panels A and C, 50 μm; panel B, 100 μm.

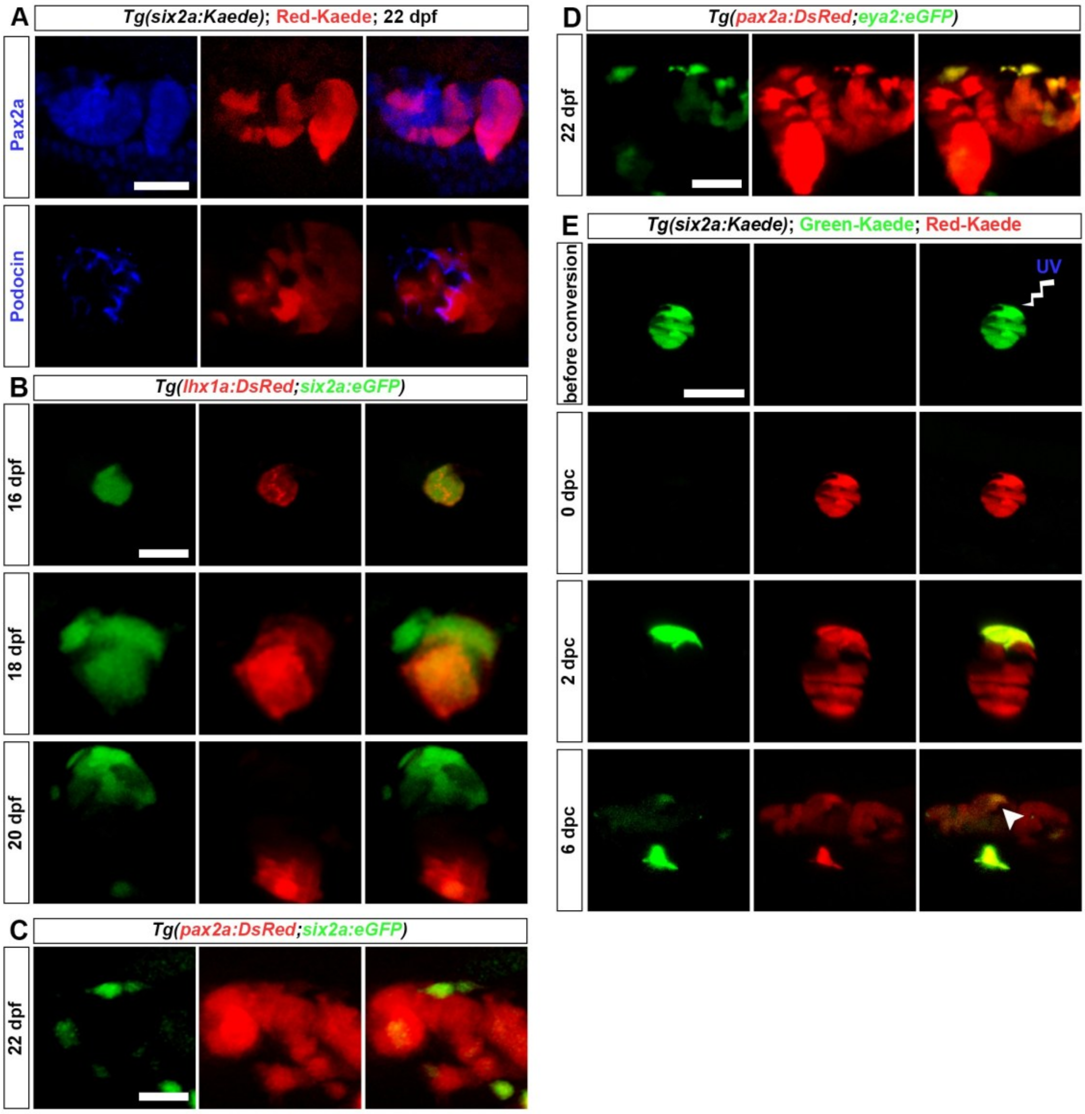

fig. S8. RSC aggregates give rise to nephron epithelial cells and nascent RSCs. **(A)** Confocal images showing Pax2a or Podocin immunofluorescence, demonstrating that photoconverted *six2a:Kaede*-labeled RSC aggregates differentiated into renal tubular epithelial cells (Pax2a$^+$) and podocytes (Podocin$^+$). **(B)** Confocal images of *Tg(lhx1a:DsRed;six2a:eGFP)* zebrafish at 16, 18, and 20 dpf, illustrating the process by which *six2a*$^+$ cells detach from the aggregates. **(C, D)** To examine nascent RSCs, we crossed *Tg(pax2a:DsRed)* fish with *Tg(six2a:eGFP)* (C) or *Tg(eya2:eGFP)* (D). All *six2a:eGFP*$^+$ cells were also *pax2a:DsRed*$^+$ (*n* = 30 cells from 6 fish), and all *pax2a:DsRed*$^+$ cells were also *eya2:eGFP*$^+$ (*n* = 30 cells from 6 fish). **(E)** Tracking of entire photoconverted cell aggregates revealed that, in rare cases,

cells from the apical region of the aggregate differentiated into nephron epithelial cells. The green cells indicated by the arrowhead were derived from the apical cell. Scale bars, 50 μm.

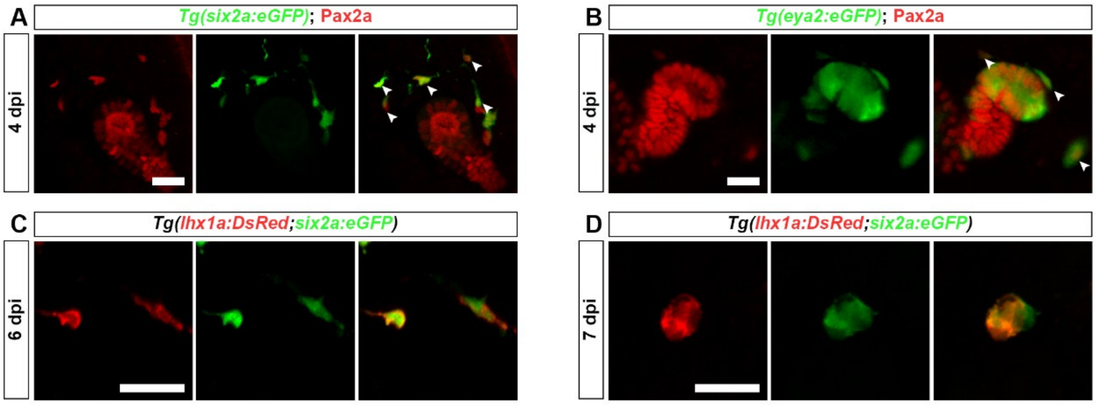

fig. S9. Molecular markers of nascent RSCs in the adult kidney. (A, B) Confocal images showing Pax2a immunofluorescence in *Tg(lhx1a:DsRed;six2a:eGFP)* (A) and *Tg(lhx1a:DsRed;eya2:eGFP)* (B) zebrafish kidneys at 4 dpi following AKI, indicating that nascent RSCs are Pax2a^{+}*eya2*$^{+}$*six2a*$^{+}$. Arrowheads indicate nascent RSCs. **(C, D)** Confocal images of *Tg(lhx1a:DsRed;six2a:eGFP)* zebrafish kidneys at 6 dpi (C) and 7 dpi (D) following AKI, showing that nascent RSCs begin expressing *lhx1a* at 6 dpi and cluster to form cell aggregates by 7 dpi. Scale bars in panels A, B, and D: 50 μm; panel C: 20 μm.

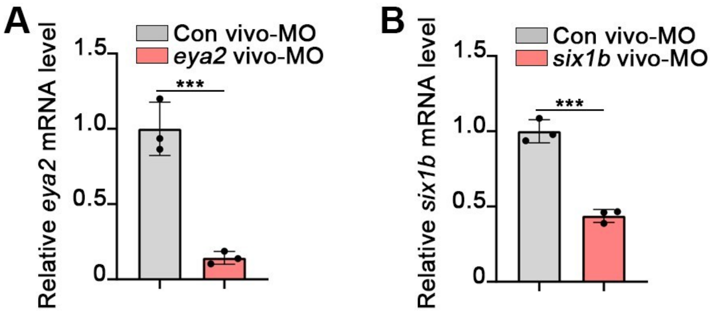

fig. S10. Efficiency verification of *eya2* vivo-MO and *six1b* vivo-MO. (**A**) qRT-PCR analysis of *eya2* in 5 dpi kidneys after administration (at 2 and 4 dpi) of *eya2* vivo-MO or Con vivo-MO following AKI. (**B**) qRT-PCR analysis of *six1b* in 5 dpi kidneys after administration (at 2 and 4 dpi) of *six1b* vivo-MO or Con vivo-MO following AKI.

movie S1. The process of *six2a* expression in RSCs. The time-lapse recording captures the gradual increase in *six2a:eGFP* expression in RSCs within *Tg(six2a:eGFP;cdh17:DsRed)* zebrafish from 86 hpf to 90.5 hpf. The *six2a:eGFP* expression is observed in RSCs situated between the renal tubules. Images were taken at 1.5-hour intervals. Scale bar, 50 μm.

movie S2. Rotating view of the 89 hpf time point shown in movie S1. The image is first rotated along the anterior–posterior axis, followed by rotation along the dorsal–ventral axis. *six2a*$^{+}$ cells are located in close proximity to the pronephric tubules. Scale bar, 50 μm.

movie S3. The maturation and migration of RSCs. The time-lapse recording captures the maturation and migration of RSCs within *Tg(six2a:eGFP;cdh17:DsRed)* zebrafish from 96 hpf to 108 hpf. During this period, *six2a:eGFP*$^{+}$ RSCs migrate from between the renal tubules to a position approximately 20 μm above the tubules, accompanied by a significant increase in cell volume. Images were taken at 1.5-hour intervals. Scale bar, 50 μm.

Table S1. scRNA-seq cluster markers. Related to Fig. 1A-C. Markers shown for each cluster were derived from scRNA-seq of zebrafish kidney cells (Fig. 1A). Selection was performed using the Seurat "FindAllMarkers" function with the parameter logFC.threshold = 0.25.

Table S2. Top markers of RSCs sub-clusters. All markers identified for each cluster were obtained from scRNA-seq of RSCs as shown in Fig. 1F. Markers were selected using the Seurat "FindAllMarkers" function with following specified parameters: logFC.threshold = 0.25.

Table S3. Reagents and resources. Detailed information on antibodies, chemicals, critical commercial assays, zebrafish lines, cell lines, and software used in this study.

Table S4. Oligonucleotides sequences. Oligonucleotides sequences used for CRISPR/Cas9 sgRNAs, genotyping, qPCR and plasmid construction.

Table S3. Reagent or Resource

REAGENT or RESOURCE	SOURCE	IDENTIFIER
**Antibodies**		
Anti-Pax2a, rabbit polyclonal	Abcam, ab229318; Dilution, 1:200	
Anti-β-actin, rabbit monoclonal	HUABIO, ET1701-80; Dilution, 1:5000	RRID:AB_2943481
Anti-DYKDDDDK Tag Rabbit monoclonal	Cell Signaling Technology,14793; Dilution, 1:100 for immunoprecipitation (IP)	RRID:AB_2572291
Anti-DDDDK-tag mouse monoclonal	MBL International, M185-3; Dilution, 1:2000 for western blotting (WB)	RRID:AB_591224
Anti-Cdh1 rabbit polyclonal	GeneTex, GTX125890; Dilution, 1:200	RRID:AB_11167551
Anti-Cdh2 rabbit polyclonal	GeneTex, GTX125885; Dilution, 1:200	RRID:AB_2885609
Anti-HA-tag rabbit monoclonal	Cell Signaling Technology, 3724, Dilution, 1:100 for IP, 1:2000 for WB	RRID:AB_1549585
Anti-GFP biotin-goat polyclonal	Abcam, ab6658; Dilution, 1:400	RRID:AB_305631
Anti-Podocin rabbit polyclonal	This study; Dilution, 1:200	
Anti-Eya2 rabbit polyclonal	This study; Dilution,1:2000	
HRP-conjugated goat Anti-mouse IgG(H+L), goat polyclonal	Protein-tech, SA00001-1; Dilution, 1:5000	RRID:AB_2722565
HRP-conjugated goat anti-rabbit IgG, goat polyclonal	Protein-tech, SA00001-2; Dilution, 1: 5000	RRID:AB_2722564
Donkey anti-Rabbit IgG (H+L), Alexa fluor 647, donkey polyclonal	Thermo Fisher Scientific, A-31573; Dilution, 1:500	RRID:AB_2536183

Donkey anti-Goat IgG (H+L)， Alexa fluor 488, donkey polyclonal	Thermo Fisher Scientific, A-11055; Dilution, 1:500	RRID:AB_2534102
Donkey anti-Goat IgG (H+L)， Alexa fluor 647, donkey polyclonal	Abcam, ab150131; Dilution, 1:500	RRID:AB_2732857
Goat anti-rabbit IgG (H+L), Alexa fluor 633, goat polyclonal	Thermo Fisher Scientific, A21070; Dilution, 1:500	RRID:AB_2535731
Sheep anti-digoxigenin-peroxidase antibody, Sheep polyclonal	Roche, 11207733910; Dilution, 1:500	RRID:AB_514500
**Chemicals**		
iCRT 14	MCE, HY16665	
BIO	MCE, HY-10580	
Anti-DYKDDDDK Affinity Beads	Smart-Lifesciences, SA042001	
Lipo8000 transfection reagent	Biyotime, C0533	
rProtein A/G Beads 4FF	Smart-Lifesciences, SA032005	
**Critical Commercial Assays**		
Western ECL Substrate	BIO-RAD, 1705060	
Prime script II 1st strand cDNA synthesis Kit	Takara, 9767	
TB Green Premix EX Taq II	Takara, RR820A	
One-Lumi™ Firefly Luciferase Assay Kit	Beyotime, RG055M	
ChIP assay kit	Beyotime, P2080S	
BCA Protein Assay Kit	Biosharp, BL521A	
Click-iT Plus EdU Alexa Fluor 647 Imaging Kit	Invitrogen, C10640	
TSA Plus Cy3 system	PerkinElmer, NEL744001KT	
TSA Plus Fluorescein	PerkinElmer, NEL741001KT	
**Zebrafish lines**		

TgKI(six2a:p2a-Gal4-VP16)	This study	
TgKI(osr1:p2a-Gal4-VP16)	This study	
TgKI(lhx1a:p2a-Gal4-VP16)	This study	
TgKI(eya1:p2a-Gal4-VP16)	This study	
TgKI(eya2:p2a-Gal4-VP16)	This study	
Tg(pax2a:DsRed)	This study	
eya2$^{-/-}$	This study	
fzd9b$^{-/-}$	China Zebrafish Resource Center, catalog ID: CZ370	
Tg(UAS:eGFP)	Laboratory	
Tg(UAS:Kaede)	Laboratory	
Tg(lhx1a:DsRed)	Laboratory	
Tg(cdh17:DsRed)	Laboratory	
**Cell line**		
HEK-293T	Laboratory	
**Software**		
Seurat 4.3.0 for R	doi: 10.1016/j.cell.2021.04.048	RRID:SCR_016341
R 4.2.3	https://www.r-project.org/	RRID:SCR_001905
RStudio	https://posit.co/	RRID:SCR_000432
Excel 2019	Microsoft, version office home and student	RRID:SCR_016137
ImageJ for Windows, V 1.8.0	National Institutes of Health	RRID:SCR_001935

Table S4. Oligonucleotides

Species	Gene	Name	Sequence (5'-3')
zebrafish	*eya1*	knockin_gRNA	GGCCCAGGTCAGTACCTTAA
zebrafish	*eya1*	all-arm_F	CGATCAGCAGGTAATACGATCACCTC
zebrafish	*eya1*	all-arm_R	CTGTATTTATAAACTGCTCACCAATGTC
zebrafish	*eya1*	eya1:p2a-Gal4_F	TCTGTAGCAGACGGCTCTCTAGACGGAAGCGGAGCTACTAACTTCAGC
zebrafish	*eya1*	eya1:p2a-Gal4_R	TTACCTGATCCTGAGAAGCTCCGGATCCAGACATGATAAGATACATTG
zebrafish	*eya1*	knockin_ID_F	CGTTAAGTCCATGACGTGCAACAGG
zebrafish	*eya1*	Probe_F	GTAGTCTATGGAAATGCAGGATCTAGC
zebrafish	*eya1*	Probe_R	GGAGAAGTGTAAGGGCTACTGTTGTAATAC
zebrafish	*eya1*	knockin_ID_R	GAGGCATATCAGTCTCCACTGAAGC
zebrafish	*eya1*	qRT-PCR_F	GACGGTGTGGAGGAGGAGCAAG
zebrafish	*eya1*	qRT-PCR_R	GGGGCCGTCACAGTGCTAC
zebrafish	*eya2*	knockin_gRNA	GGGGGCGTCCCGCTGAAGTT
zebrafish	*eya2*	left-arm_F	CAGTCTGTAGTTTCATCATTACACA
zebrafish	*eya2*	left-arm_R	ACCCTGGAAAATTCCAGAAGAA
zebrafish	*eya2*	right-arm_F	GCGAACAGCATCACGGGCTCAAC
zebrafish	*eya2*	right-arm_R	CTTGTGTGTAAGCGAGGCACAAC
zebrafish	*eya2*	knockin_ID_F	GTCTGACAACCACCAGTGAACCAAGG
zebrafish	*eya2*	knockin_ID_R	GAGGCATATCAGTCTCCACTGAAGC
zebrafish	*eya2*	knockout_gRNA	GGGGGCGTCCCGCTGAAGTT
zebrafish	*eya2*	knockout_ID_F	CAGTGCACTACAGAGATTCGG
zebrafish	*eya2*	knockout_ID_R	TGACTGTTGCACTTTTCCCCA
zebrafish	*eya2*	qRT-PCR_F	GAGGAGCGTTTCCCATCGAGAAC
zebrafish	*eya2*	qRT-PCR_R	CTCCTCTTCAATACCGTCTCCTACG
zebrafish	*eya2*	FISH-probe_F	CATTCACTCCACAACAGCACTG
zebrafish	*eya2*	FISH-probe_R	CCTGAGAGTGTGTGCATGTG
zebrafish	*eya2*	3HA-eya2_F	ATGGCAGCTTACGGACAGACGCAGTACAG
zebrafish	*eya2*	3HA-eya2_R	CTAGAGGTAATCCAGCTCTAGTGCATGACTC
zebrafish	*eya2*	7K-promoter_F	CTTACGACCGCTGTTTTTTATGGCGTTG
zebrafish	*eya2*	7K-promoter_R	TTCGTCCCGCTGCGCGTGATTGTATG

zebrafish	*eya2*	Δ_F	CTACTTGGTTTTATTAAAAAAAGTGATG CGCTGCCTTC
zebrafish	*eya2*	Δ_R	CATCACTTTTTTTAATAAAACCAAGTAGT AAAGTAGTGACTCCCAG
zebrafish	*eya2*	EMSA-probe_F	Digoxigenin-CACCACTGATAAAAACTATTAAAGATC
zebrafish	*eya2*	EMSA-probe_R	Digoxigenin-GTGAACGCATTTAAAGAAGGCAG
zebrafish	*six2a*	knockin_gRNA	GGGAAAAGTCTTAAAGCCCG
zebrafish	*six2a*	left-arm_F	ACGACAACGCGACCGAGCAGC
zebrafish	*six2a*	left-arm_R	GAGCCAAGGTCGACCAAGTTTG
zebrafish	*six2a*	right-arm_F	TAAACGTGGACCCTTTCAAAAG
zebrafish	*six2a*	right-arm_R	GCCTAATGTGCTCGATAACAAG
zebrafish	*six2a*	knockin_ID_F	GGAGAAAAGGGAACTAGCTGAG
zebrafish	*six2a*	knockin_ID_R	GAGGCATATCAGTCTCCACTGAAGC
zebrafish	*six2a*	Pobe_F	GTCTATGCTTCCAACATTCGGCTTTAC
zebrafish	*six2a*	Probe_R	CTCATTTGTTTGTGTTTATACGCATCTC GC
zebrafish	*lhx1a*	knockin_gRNA	TGGCAGCCAGGGAAGCCGCA
zebrafish	*lhx1a*	left-arm_F	GCAAAATGACGAGCAGATACACTAATA G
zebrafish	*lhx1a*	left-arm_R	CTGTAGCTGGTCTTGAGAATCTGG
zebrafish	*lhx1a*	right-arm_F	GACGATGTCAAGGATGCGG
zebrafish	*lhx1a*	right-arm_R	GCCAAGACTCCCATCGTTCTCACC
zebrafish	*lhx1a*	knockin_ID_F	CTCCAGCGATTCAACAGCTTTACAC
zebrafish	*lhx1a*	knockin_ID_R	GAGGCATATCAGTCTCCACTGAAGC
zebrafish	*osr1*	knockin_gRNA	GGCAGGCAAATGCAGCCCGT
zebrafish	*osr1*	left-arm_F	AAACAGGTTTTCTGGGGAGCCCATAAT G
zebrafish	*osr1*	left-arm_R	GATATCACAAGTGTACGGCCGCTC
zebrafish	*osr1*	right-arm_F	GATATCTGTCATAAGGCCTTTAGGAGG
zebrafish	*osr1*	right-arm_R	GTGACAGAAACCCTTTCCGCACTCTTG GC
zebrafish	*osr1*	knockin_ID_F	CAGGGCTGTAGTCGAAAGTGCAAAC
zebrafish	*osr1*	knockin_ID_R	GAGGCATATCAGTCTCCACTGAAGC

zebrafish	*pax8*	Probe_F	GTCATGGGGGTCTTAATCAACTAGGAGG
zebrafish	*pax8*	Probe_R	GTCTGGTGGAGGGTTAGAGATGATCATAG
zebrafish	*pax2a*	Promoter_F	GGAGCCATGGCTATTGATTTAG
zebrafish	*pax2a*	Promoter_R	GGGATAGCAAAGGAAGGAAAGATAATC
zebrafish	*wnt4a*	Probe_F	ATGTCATCGGAGTATTTGATAAGG
zebrafish	*wnt4a*	Probe_R	AAGCAGCATCACAGGAACAGC
zebrafish	*wnt4a*	qRT-PCR_F	CTGGAGAGCGTTCCTGTGTTTGG
zebrafish	*wnt4a*	qRT-PCR_R	GTCACATCCACACTTGTCCAGC
zebrafish	*slc20a1a*	qRT-PCR_F	GGTGCACAGACAGACATGTCTGATG
zebrafish	*slc20a1a*	qRT-PCR_R	GTCTCAAAGATGGTAGCCAGGATGC
zebrafish	*six1b*	3Flag_six1b_F	ATGTCAATGTTGCCTTCTTTCGGGTTTACGCAGG
zebrafish	*six1b*	3Flag_six1b_R	TTAAGAGCCTAGGTCCACTAGACTTGAAG
zebrafish	*six1b*	Probe F	ATGTCAATGTTGCCTTCTTTCGGG
zebrafish	*six1b*	Probe R	GGTTTGTCTTCTGACCAGTTTAACGACTTGGC
zebrafish	*six1b*	qRT-PCR_F	GACTGACTACCACACAAGTGAGCAAC
zebrafish	*six1b*	qRT-PCR_R	GACAGCTGGTTCTGTTTGTTAGCG
zebrafish	*lef1*	3Flag_lef1_F	ATGCCGCAGTTGTCAGGTGGAG
zebrafish	*lef1*	3Flag_lef1_R	TCAGATGTACGCCGTTTTCATTCTCTG
**Vivo-MO**			
zebrafish	*eya2*	eya2 vivo-MO	CGTCTGTCCGTAAGCTGCCATGAGC
zebrafish	*six1b*	six1b vivo-MO	CGCTTAATTACCTTTCTTTCGCCTC
		Con vivo-MO	TCTGTGGATGTCTTGCTCTTCCAGG
